# Pharmacological inhibition of PLK1/PRC1 triggers mitotic catastrophe and sensitizes lung cancers to chemotherapy

**DOI:** 10.1038/s41419-025-07708-8

**Published:** 2025-05-12

**Authors:** Pingping Li, Yufei Zhao, Minghan Lu, Chengfei Chen, Yongkun Li, Lingling Wang, Shulan Zeng, Yan Peng, Hong Liang, Guohai Zhang

**Affiliations:** 1 Key Laboratory for Chemistry and Molecular Engineering of Medicinal Resources (Ministry of Education of China), Collaborative Innovation Center for Guangxi Ethnic Medicine, Guangxi Key Laboratory of Chemistry and Molecular Engineering of Medicinal Resources, School of Chemistry and Pharmaceutical Sciences, Guangxi Normal University, Guilin, China; 2 School of Comprehensive Health Management, Xihua University, Chengdu, China; 3 Joint Medical Research Center of Guangxi Normal University & Guilin Hospital of Chinese Traditional and Western Medicine, Guilin, China

**Keywords:** Drug development, Target identification

## Abstract

Polo-like kinase 1 (PLK1) signaling drives tumor malignancy and chemotherapy resistance, which is an unmet clinical need. Recruiting PLK1 to the central spindle during anaphase is necessary for its function in promoting cancer cell proliferation, which is achieved by binding to microtubule-associated protein regulating of cytokinesis (PRC1) located in the spindle. However, the role of PLK1/PRC1 signaling in chemotherapy resistance is unknown. In this study, we identified a small molecule **B4** which inhibited PLK1/PRC1 signaling through disrupting the formation of PLK1/PRC1 protein complexes. In the presence of blocking PLK1/PRC1 signaling, enhanced sensitivity of drug-resistant tumors to traditional chemotherapy was found. Suppression of PLK1 activity by **B4** inhibited disease progression in allograft models, and combination with cisplatin elicited dramatic regression of drug-resistant tumors. Our findings provide a promising strategy to target the PLK1 signaling cascade and demonstrate a potential modality to enhance sensitivity to chemotherapy in non-small cell lung cancer (NSCLC).

## Introduction

Drug resistance is still a major cause of failure in cancer treatment, which always leads to tumor recurrence [1–3]. The mechanisms of chemotherapy resistance are multifactorial and complex [4–7], and many drugs lose efficacy through acquired drug resistance, leading to relapse of the malignancy after clinical response [8–10]. The past researches mainly focused on the genetic drivers of drug resistance, by identifying pre-existing gene alterations or the acquired new mutations during treatment [2, 3]. Wherein, dysregulation of protein kinase is emerging as a critical driving factor for chemotherapy resistance [11**–**14]. Because of the critical role of drug resistance in cancer treatment failure, identifying novel targets for chemotherapy resistant-patients is of great importance.

PLK1 is an evolutionarily conserved Ser/Thr kinase, which plays an important role in the regulation of various cellular events such as mitosis, cytokinesis and DNA replication/repair [15, 16]. PLK1 is overexpressed in various malignant tumors, which is associated with poor prognosis of cancer patients [17, 18]. Moreover, PLK1 has been found to be related to the chemoresistance of several chemotherapeutic drugs, including cisplatin, paclitaxel, gemcitabine and so on [14, 19–21]. Given the overexpression features of PLK1 in tumors and its potential role in chemoresistance, targeting PLK1 pathway is an attractive strategy for overcoming drug resistance in cancer treatment.

The PLK1 protein consists of an N-terminal catalytic kinase domain (KD) and two polo box domains (PBD) located at its C-terminus, which is responsible for substrate recognition and subcellular localization [22]. The spatiotemporal regulation of PLK1 activity is necessary for its function in mitosis regulation, which is achieved by binding to interacting proteins with different subcellular localization [23**–**26]. After its activation, the phosphorylated PRC1 is firstly recognized by PLK1 PBD [23, 24, 27], and PLK1 phosphorylates PRC1 subsequently to produce specific docking sites on PRC1 to interact with PLK1, thereby recruiting PLK1 to the spindle and promoting mitotic progress. Pharmacological inhibition of PLK1 kinase activity could prevent the formation of the specific docking site on PRC1, but whether this action could completely block the interaction between PLK1 and PRC1 is unclear, as CDK1 could also phosphorylate PRC1 to produce phosphorylation sites for binding with PLK1 PBD [27]. Furthermore, due to the evolutionary conservation of ATP binding sites, kinase domain (KD) inhibitors of PLK1 targeting this site promiscuously inhibit other PLKs kinases (PLK2, PLK3, PLK4, PLK5), which makes these inhibitors pan-selective and could exhibit dose-limiting toxicity [22].

In this study, we report an alternative inhibitor targeting PLK1 PBD which disrupted the formation of the PLK1/PRC1 signaling complex and inhibited the spatiotemporal coordination of PLK1 activity, resulting in mitotic catastrophe and enhanced sensitivity to chemotherapy in NSCLC. Therapeutically, suppression of PLK1 by this inhibitor attenuated NSCLC tumor growth and sensitized tumors to cisplatin in allograft tumor models. Our findings provide an alternative strategy and a new modality to target the PLK1 pathway for enhancing the sensitivity of NSCLC to chemotherapy.

## Materials and methods

### Materials and cell lines

Anti-PRC1 (15617-1-AP) was purchased from Proteintech. Anti-phospho-PLK1(T210) (ab155095) antibodies were purchased from Abcam. Anti-PLK1 (AF7776), anti-Cyclin B1 (AF1606) and anti-CDK1 (AF1516) were obtained from Beyotime (Shanghai, China). Anti-DDK (FLAG) monoclonal antibody (TA50011-100) was purchased from origene. Anti-GAPDH (M1211-1) was purchased from HUABIO. Anti-rabbit and anti-mouse secondary antibodies were obtained from ZSGB-BIO. 1×PBS (pH7.2–7.4, 0.01 M, cell culture, P1020), crystal violet (C8470), RNase A (R1030), propidium iodide (PI, P8080) and NP40 (N8030) were purchased from Solarbio (Beijing, China). Recombinant PLK1 protein (0.25 μg/μL, 81429) was obtained from Active mofit. A549/DDP cell lines (human lung cancer with acquired cisplatin-resistant) was obtained from Pricella. HCC-827 (human lung cancer cells), NCI-H1975 ((human lung cancer cells) and A549 (human lung cancer cells) cell lines were provided by Stem Cell Bank, Chinese Academy of Sciences. The HCC-827, NCI-H1975, A549 and A549/DDP cells lines were cultured in RPMI 1640 medium (GIBCO-BRL, Grand Island, NY) supplemented with 10% fetal calf serum (FCS).

### Bioinformatics data analysis

We downloaded the RNA-seq data and clinical phenotype information of the LUAD-related tumor and the adjacent normal tissues from The Cancer Genome Atlas (TCGA) database (https://www.cancer.gov/ccd/research/genome-sequencing/tcga) or GEO database. The grouping information adopts the median method, with high expression above the median and low expression below the median. We further did the correlation analysis between the PLK1 gene and the disease stage.

### Kaplan–Meier plotter analysis

The Kaplan–Meier plotter (http://kmplot.com/analy sis/) [28] is a web tool that provides gene survival analysis in various cancer types. The LUAD specimens were assigned to low and high PLK1 expression groups, the overall survival (OS) analysis of PLK1 in LUAD was conducted.

### Molecular docking

The crystal structure of PLK1 protein (PDB: 3FVH, 1.58 A°) was downloaded from Protein Data Bank (http://www.rcsb.org/pdb/). The three-dimensional structure of **B4** was obtained via ChemBio3D Ultra software. PYMOL 2.3.4 software and AutoDock Tool was used to modify the receptor protein with water removal, ligand removal, hydrogen addition and charge balancing, and the Grid Option tool was opened with the Grid Box size command under the Grid program to adjust the number of grid points in each direction, the center of the binding pocket and the spacing of the grid points. Grid Box size was set as 60×60×60. Molecular docking of PLK1 to **B4** was performed using Autodock 4, and the result was output as an affinity.

### Surface plasmon resonance (SPR) assay

SPR experiments were performed at 25 °C with Biacore T200 instrument (Cytiva). Recombinant PLK1 protein was prepared in sodium acetate buffer (pH 5.0) and then immobilized on the Fc2 (16000 response unit) surfaces of a sensor chip CM5 at a concentration of 20 μg/ml using standard EDC/NHS coupling chemistry followed by ethanolamine deactivation of the surfaces. The binding affinity of **B4** for immobilized PLK1 was determined by injecting **B4** solutions with different concentration from 0 to 10 μM in PBS-DMSO buffer (10 mM PBS (pH 7.2–7.4), 136 mM NaCl, 2.6 mM KCl, and 5% DMSO) over the surfaces at a constant flow rate of 30 μL/min. Data was analyzed using Biacore analysis software and Fig.s generated using Origin 8.0. Results are the mean of two independent experiments.

### Cellular thermal shift assay (CETSA)

For intact cell CETSA, the A549/DDP cells were treated with DMSO or **B4** (5 μM) for 2 h, then the collected cells were divided into 13 equal volumes, one of them was used as the input sample and others heated for 3 min at specified temperatures as the method in the CETSA in cell lysates. Then, the cells were frozen- thawed twice using liquid nitrogen. Samples were centrifuged and supernatants were analyzed by western blotting. For the cell lysates CETSA, A549/DDP cells were collected with precooling PBS containing 1% PMSF and repeatedly freeze-thawed with liquid nitrogen. After centrifugation, the supernatant was collected and divided into two groups and then incubated with 20 μM **B4** or DMSO at 25 °C for 2 h. Afterwards, the operation was performed as for CETSA in intact cells.

### Ligand-observed NMR binding experiments

Ligand observation T1ρ NMR experiments were performed to investigate the ligand-protein interactions. Samples containing 200 μM **B4** and 200 μM **B4** mixed with 5 μM or 10 μM of PLK1 protein. Compound and protein were solubilized in phosphate buffer (20 mM sodium phosphate, pH 7.4, 100 mM NaCl, 10% DMSO) and then detected by Bruker Avance Neo 600 MHz spectrometers at 25 °C and analyzed by MestReNova.

### Plasmid vector construction and transfection

The DNA sequences encoding PLK1 KD and PLK1 PBD were clustered into the restricted BamHI site with a modified pcDNA 3.1 (+) from 3Flag-CMV-EGFP-tag. The construction of plasmid vector was performed by Hanbio Biotechnology. When the cells reached 90% confluence, the culture medium was replaced with Opti-MEM. Then the plasmid (2.5 μg/well) was transfected into A549/DDP cells via Lipofectamine 2000 reagent (6 μL/well) according to the manufacturer’s instructions. The transfection efficiency was verified by fluorescence microscopy after 24 h. Finally, cell lysates from A549/DDP cells were exposed to 10 μM **B4** for 2 h at 37 °C and then immunoprecipitated according to the protocol of the Co-IP assay. After treatment with ultrasound for 1 min, the eluted samples were detected by HR-MS.

### Co-immunoprecipitation (Co-IP)

A549/DDP cells were treated with **B4** (6 μM) at 48 h, lysed with lysis buffer and then followed by immunoprecipitation according to the protocol of the Co-IP assay (Thermo, 26149). Lysates were immunoprecipitated with anti-PRC1-coupled agarose beads (rabbit antibody) for 2 h. Samples were washed 3 times with lysis buffer, one time with Last wash buffer and then bound proteins were eluted with elution buffer for further examination.

### In vivo xenograft model assay

Nude mice (6 weeks old) were purchased from Hunan SJA Laboratory Animal Co., Ltd. (Changsha, China). Human lung cancer A549/DDP and A549 cells in 200 μL of serum-free culture medium were subcutaneously injected into the upper flank region of the nude mice. After the tumor growth of mice, mice were randomly assigned to four groups: the control group (5% DMSO, 30%PEG-300 and 65% saline), 30 mg/kg **B4** treated group (dissolved in 5% DMSO, 30%PEG-300 and 65% saline), 1 mg/kg DDP treated group (dissolved in saline solution) and **B4** & DDP co-treatment group. Mice were then administered with or without **B4** and DDP via intraperitoneal injection every day for 14 days. The body weight and tumor size of the mice were measured three times a week. Tumor volumes was measured the length (l) and width (w) using a Vernier caliper and calculated the volume (*V* = lw^2^/2). All animal experiments were conducted in accordance with the guidelines of the Institutional Animal Care and Use Committee and were approved by the Animal Ethics Committee of Guangxi Normal University.

### Statistical analysis

Statistical analyses were performed using GraphPad Prism 9 software. Data are expressed as means ± SD. Data were considered statistically significant at P < 0.05. All data are shown as mean ± standard deviation (SD) using two-tailed Student t tests and one-way ANOVA with Bonferroni multiple comparison post-test.

## Results

### Overexpression of PLK1 is associated with poor prognosis in NSCLC

**Fig. 1:**
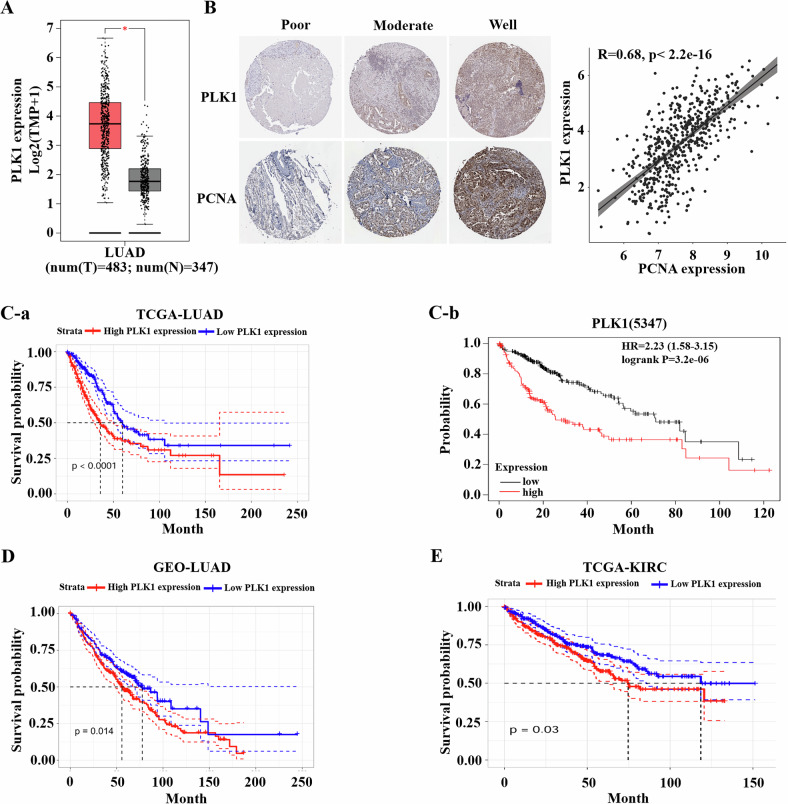
High expression of PLK1 in NSCLC correlates with poor prognosis. **A** mRNA levels of PLK1 in NSCLC tissues and normal tissues, *P < 0.05. **B** Immunohistochemical analysis to detect the protein expression of PLK1 and proliferating cell nuclear antigen (PCNA) in tumor tissues of NSCLC patients with different degrees of differentiation (left), and the correlation study between PLK1 and PCNA expression in tumor tissues of NSCLC patients (right). **C-a** Correlation analysis between PLK1 and overall survival (OS) of NSCLC patients in TCGA database. **C-b** Correlation analysis between PLK1 and OS in patients with advanced NSCLC in the Kaplan-Meier mapper database. **D** Correlation analysis between PLK1 and OS in NSCLC patients in the GEO database. **E** Correlation analysis between PLK1 and OS in KIRC patients in the TCGA database.

To investigate the clinical significance of PLK1 expression in NSCLC, we firstly analyzed the expression profiles of PLK1 in 483 adenocarcinoma (LUAD) samples and 347 normal lung samples from The Gene Expression Profiling Interactive Analysis data. The result showed that the expression of PLK1 in LUAD tissue was higher than that in normal tissue (Fig. 1A). Furthermore, the expression of PLK1 increased with the progression of NSCLC (Fig. 1B). Compared with moderately differentiated NSCLC, the expression of PLK1 protein was significantly increased in highly differentiated NSCLC in the human protein atlas (HPA) database (Fig. 1B, left). There was a notable positive correlation between the expression of PLK1 and the expression of proliferating cell nuclear antigen (PCNA) (R = 0.68) in tumor tissue (Fig. 1B, right). Next, we examined the prognostic value of PLK1 in LUAD. A high PLK1 expression level was correlated with a decrease in overall survival (OS) in TCGA (Fig. 1C-a) or Kaplan-Meier plotter databases (Fig. 1C-b). PLK1 expression and prognostic value were also validated using cohorts from the GEO database (Fig. 1D). Similar result was found for Kidney Renal Clear Cell Carcinoma (TCGA-KIRC) (Fig. 1E). These data indicate that overexpression of PLK1 is associated with poor prognosis in NSCLC patients.

### PLK1 is involved in chemotherapy resistance

**Fig. 2:**
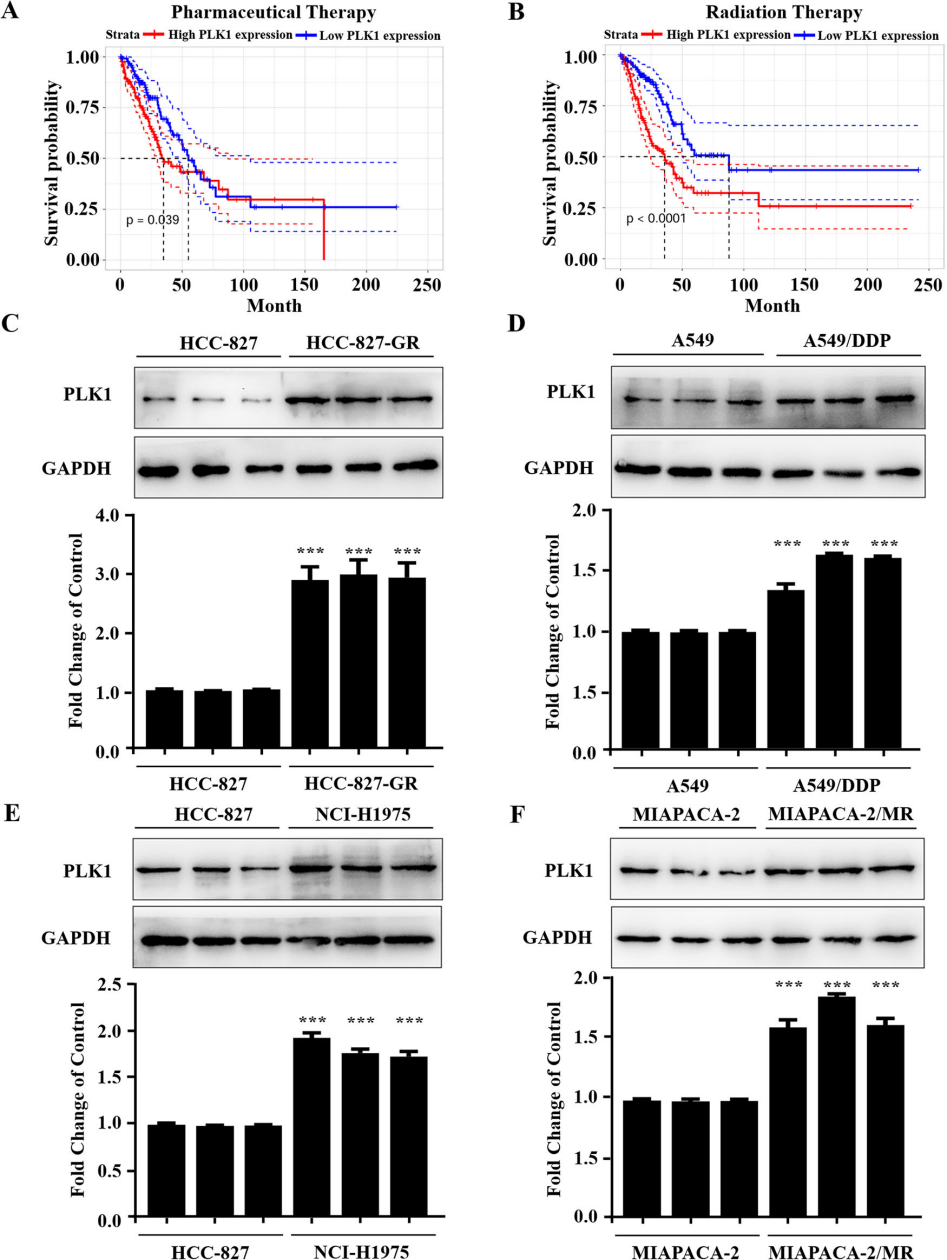
PLK1 overexpression is associated with chemotherapy resistance. **A** Correlation analysis between PLK1 expression status and overall survival rate in NSCLC patients receiving drug therapy. **B** Correlation analysis between PLK1 expression status and overall survival rate in NSCLC patients receiving radiation therapy. **C**–**F** Protein immunoblotting assay to detect PLK1 expression levels in drug-sensitive and drug-resistant cell lines, respectively: **C** HCC-827 and HCC-827/GR, **D** A549 and A549/DDP, **E** HCC-827 and NCI-H1975, **F** MIAPACA-2 and MIAPACA-2/MR. Each has the expression of GAPDH as the internal control. Protein expression was quantified by densitometry analysis using ImageJ and normalized against GAPDH expression. Data are presented as the mean ± SD. ***p < 0.001.

To explore whether PLK1 participates in tumor therapy resistance, we analyzed the impact of PLK1 expression on the survival of patients receiving chemotherapy or radiation therapy. Based on data from TCGA, we found that lung cancer patients with high expression of PLK1 exhibited poorer survival (P < 0.05) compared to those with low expression of PLK1 after receiving chemotherapy (Fig. 2A). PLK1 expression status had a more significant impact on their overall survival when patients receiving radiation therapy (P < 0.01), indicating high expression of PLK1 significantly reduced the efficacy of radiotherapy (Fig. 2B). To further verify the observation, we also checked PLK1 levels in 4 drug-resistant cell lines. Prior to testing, we examined the resistance index (RI) of the four resistant cell lines and the parental cell. A resistance index (RI) greater than 5 indicated that all four resistant cells were resistant (Fig. S1). Compared with the controls, PLK1 expression levels were greatly upregulated in drug-resistant cells (Fig. 2C–F). These results indicate that PLK1 was indeed involved in chemotherapy resistance.

### Identification of a small molecule targeting PLK1 protein specifically

**Fig. 3:**
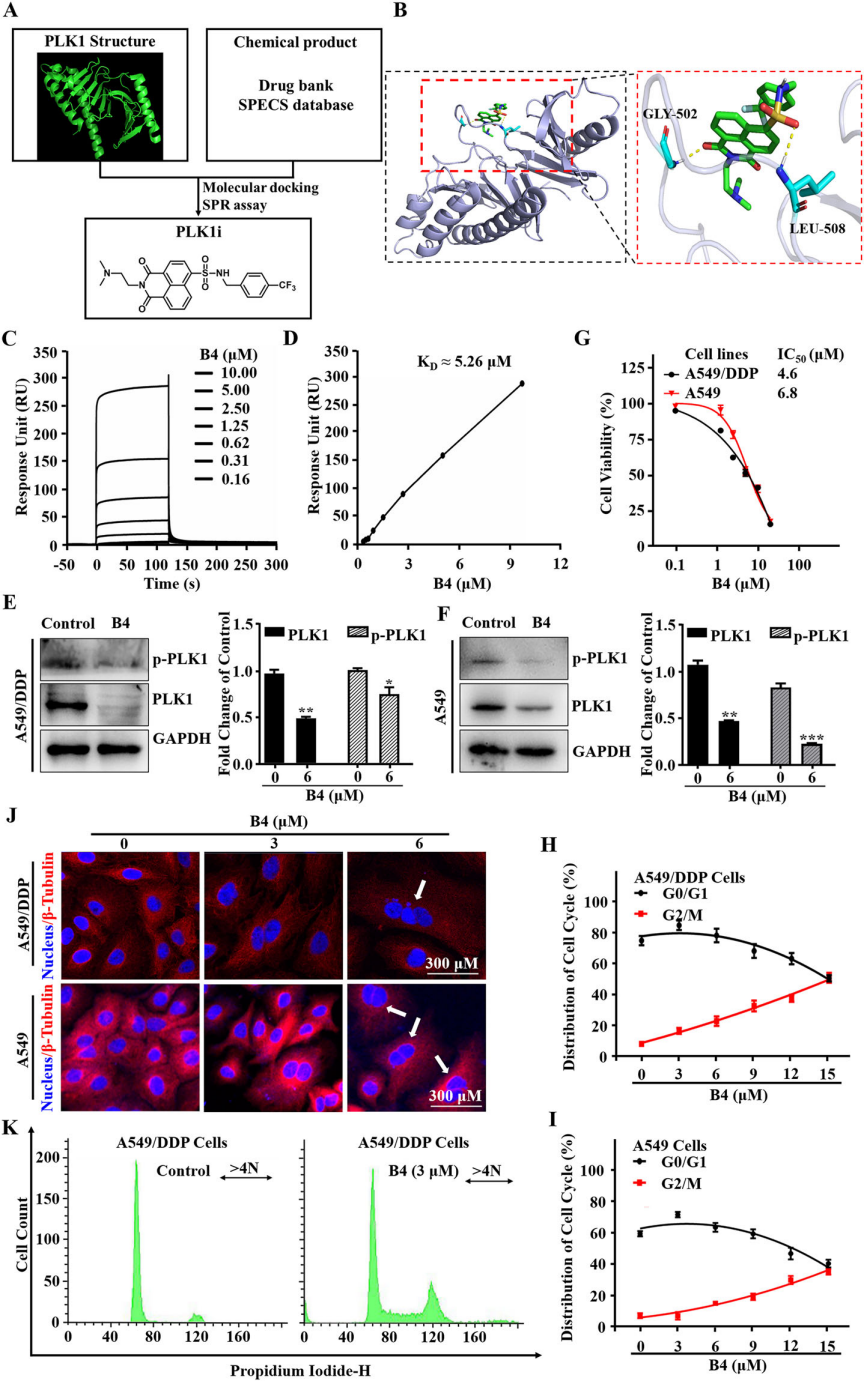
Assess of B4 as a PLK1 inhibitor. (**A**) Structure-based virtual screening workflow for PLK1-substrate interaction inhibitors. (**B**) Ribbon structure display of **B4** binding to active sites of PLK1 PBD (PDB code: 3FVH). **B4** is shown in green and protein residues involved in **B4** binding are highlighted in blue. (**C**) Interaction of compounds with PLK1 protein detected by surface plasmon resonance. **B4** binding to PLK1 protein, respectively. (**D**) Plots of the equilibrium response unit responses versus concentrations of **B4**. Results are the mean of two independent experiments. (**E**) Protein abundance of PLK1 and p-PLK1 after **B4** treatment. Lysates from A549/DDP cells after 48 h of **B4** treatment were detected by western blotting and quantified using ImageJ. Data is expressed as mean ± SD, (n = 3) **p < 0.01 and ***p < 0.001. (**F**) Protein abundance of PLK1 and p-PLK1 after **B4** treatment. Lysates from A549 cells after 48 h of **B4** treatment were detected by western blotting and quantified using ImageJ. Data is expressed as mean ± SD, (n = 3) **p < 0.01 and ***p < 0.001. **G** IC_50_ values of **B4** against A549 and A549/DDP cell lines. Data is expressed as mean ± SD, (n = 3). **H**, **I** Effect of **B4** on cell cycle in A549 cells and A549/DDP cells. After treated with **B4** for 48 h, PI staining was used to examine the cell cycle of A549 cells (**H**) and A549/DDP cells (**I**). Data is expressed as mean ± SD, (n = 3). **J** Morphological changes in the nucleus of A549/DDP and A549 cells after **B4** treatment at 48 h. Arrows indicate multinucleated cells. Images shown are representatives of three independent experiments. **K** Regulation of **B4** on cell cycle progression. PI staining was used to investigate the effects of **B4** on the cell cycle of A549/DDP cells. Data is processed through ModFit LT.

To explore the roles of PLK1/PRC1 signaling in chemotherapy resistance, we aimed to identify small molecules that directly targeted the PLK1 protein and disrupted their interactions. Considering the crucial role of PLK1 PBD in recognizing PRC1, we performed a high-throughput virtual screening based on the structural coordinates of the PLK1-phosphopeptide complex (PDB: 3 FVH) [29] to identify novel small molecules that can bind with the PBD of PLK1 as potential inhibitor. Compound **B4** was identified as a novel small molecule bound to PLK1 (Fig. 3A). We subsequently performed molecular docking analysis to determine the binding modes of **B4** to PLK1. The docking results showed that the binding sites (residues GLY502 and LEU508) were mainly localized in the secondary linkage region (L2, residues 480-502) with PB1 and PB2 (Fig. 3B), thereby limiting its movement, and inhibiting PLK1 activity [22]. In addition, we tested the binding affinity of compound **B4** to PLK1 by using surface plasmon resonance (SPR) techniques. The results showed that compound **B4** displayed a strong affinity to PLK1 with an estimated K_D_ value of 5.6 μM (Fig. 3C, D). To further verify if **B4** could inhibit PLK1 activity, we examined the PLK1 expression and PLK1 phosphorylation after treatment with **B4** in A549/DDP and A549 cells. **B4** inhibited PLK1 phosphorylation as well as decreased the abundance of PLK1 (Fig. 3E, F). Furthermore, **B4** was tested for inhibitory activity on PLK1 by an in vitro kinase assay. We observed the concentration-dependent inhibition of PLK1 with **B4**, with an IC_50_ value of 13.52 μM (Fig. S2A). Functional studies indicated that **B4** can effectively arrest the cell cycle at G2/M phases (Figs. 3H, I and S3), trigger mitotic catastrophe (Fig. 3J, K), the typical characteristics of PLK1 inhibitors [30**–**33]. These data demonstrated that the small-molecule **B4** appears to inhibit PLK1 activity in vitro. To evaluate the potential of **B4** in overcoming chemoresistance, we treated A549 and A549/DDP cells with **B4** for 48 h and assessed cell viability. As expected, we observed that **B4** treatment exhibited a potent cytotoxic activity in A549 cells with an IC_50_ value of 6.8 μM, whereas the cytotoxic activity of **B4** in drug-resistant A549/DDP cells was more profound, with an IC_50_ value of 4.6 μM (Fig. 3G).

To further validate the regulatory effect of **B4** on PLK1, we examined the PLK1 expression and PLK1 phosphorylation as well as cell cycle arrest effect after treatment with **B4** in HCC-827-GR, NCI-H1975 and MIAPACA-2/MR cells. As depicted in Fig. S4A–C, **B4** effectively inhibited the phosphorylation of PLK1 and reduce its abundance. Furthermore, **B4** caused significant G2/M phase arrest in all three cell lines (Fig. S4D–F), and sensitized the HCC-827-GR, NCI-H1975, and MIAPCA-2/MR cells to their therapeutic drugs (Fig. S4G–I).

### B4 specifically binds to PLK1 PBD and inhibits its activity in A549/DDP cells

**Fig. 4:**
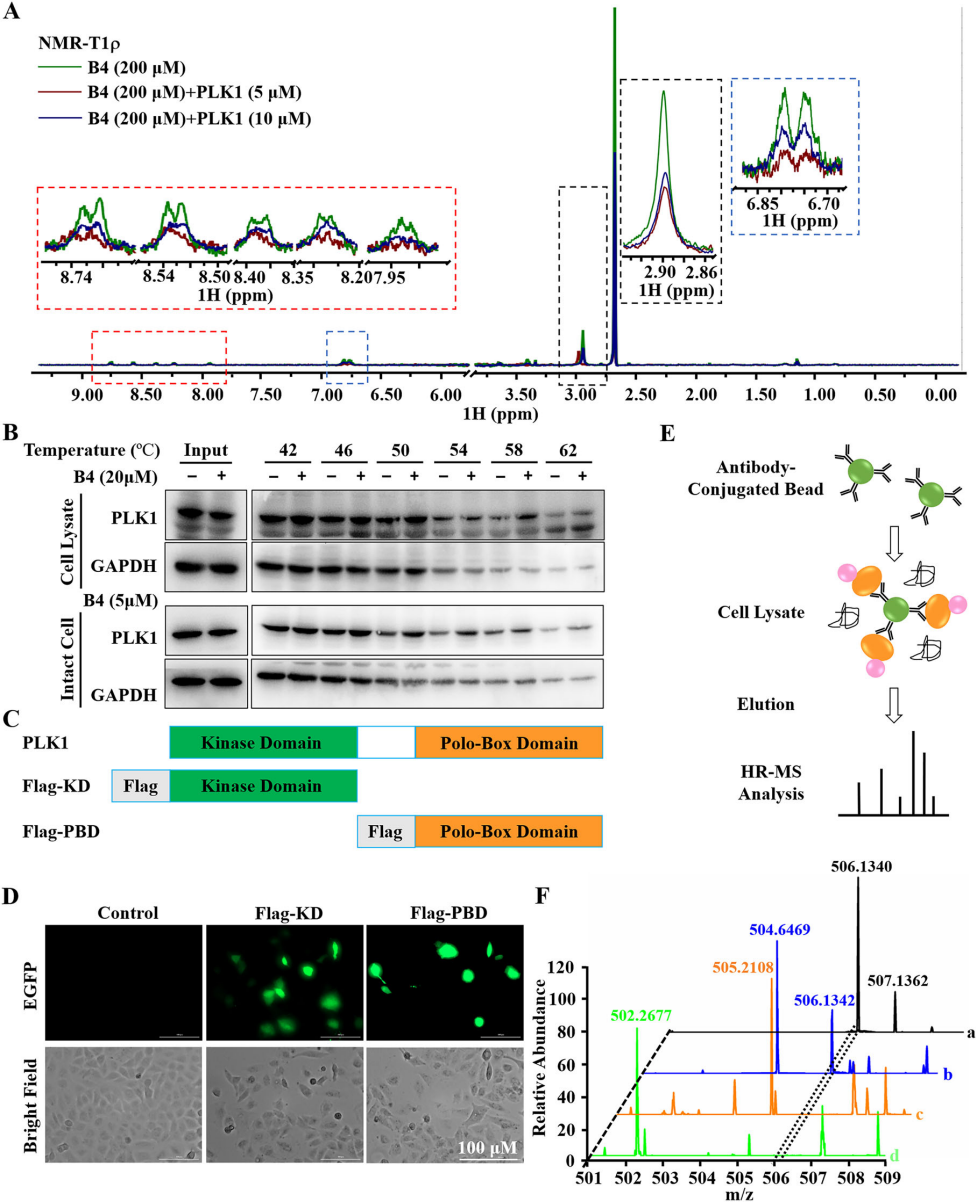
B4 binds to PLK1 PBD. **A** NMR measurements of direct binding between **B4** and PLK1. T1ρ NMR spectra for **B4** (green), **B4** in the presence of PLK1 at 5 μM (red) and 10 μM (blue). **B** Representative western blots for the stabilization of the PLK1 protein. CETSA was detected in A549/DDP cell lysates and intact cells in the presence of 20 μM and 5 μM **B4**, respectively. Each has the expression of GAPDH as the internal control. **C** 2D structural model of Flag-KD and Flag-PBD plasmids construct. **D** Images of Flag-KD and Flag-PBD plasmids transfected A549/DDP cells for 24 h were captured by Cytation 5. Images shown are representatives of three independent experiments. **E** Co-IP-MS experimental procedure. **F** Interaction between **B4** and PLK1-PBD was analyzed by HR-MS. (a) **B4** was dissolved in DMSO and detected by HR-MS (m/z: 456.1394 [M + H]^+^), black line. (b) **B4** interacted with PLK1 PBD. Whole proteins extracted from A549/DDP cells transfected with Flag-PBD plasmids were treated with **B4** (10 μМ) for 1 h, followed by immunoprecipitation with anti-Flag antibody, and finally eluted samples were examined by HR-MS after 1 min of ultrasonic treatment (m/z: 456.1370 [M + H]^+^, blue line). (c) **B4** was not combined with PLK1 KD. Whole proteins extracted from A549/DDP cells transfected with Flag-KD plasmids were treated with **B4** (10 μМ) for 1 h, followed by immunoprecipitation with anti-Flag antibody, and finally eluted samples were examined by HR-MS after 1 min of ultrasonic treatment (orange line). (d) The mass result of the negative control. Whole proteins extracted from A549/DDP cells were treated with **B4** (10 μМ) for 1 h, followed by immunoprecipitation with anti-Flag antibody, and finally eluted samples were examined by HR-MS after 1 min of ultrasonic treatment (green line).

The molecular docking and SPR analysis demonstrated that **B4** was able to bind directly to PLK1. We performed nuclear magnetic resonance (NMR) experiments to further explore the direct interactions of **B4** with PLK1 at the molecular level. The presence of 5 and 10 μM of PLK1 protein induced dose-dependent signal attenuation in the T1r NMR spectra of **B4** (Fig. 4A), suggesting that PLK1 interacted with **B4** directly.

To investigate whether the direct interaction between **B4** and PLK1 occurs in intact cells, the cellular thermal shift assay (CETSA) was carried out to measure the thermal stabilization of PLK1 in A549/DDP intact cells and cell lysates. We found that the presence of **B4** induced an obvious thermal shift of the PLK1 protein in A549/DDP intact cells and cell lysates at denaturation temperatures of 50-62 °C (Fig. 4B), indicating that **B4** directly bound to PLK1 in the cellular environment and significantly enhanced the thermal stability of PLK1.

To identify the binding region of PLK1 for **B4**, we constructed two plasmids expressing the PLK1 kinase region (Flag-KD) or PBD region (Flag-PBD) (Fig. 4C), respectively. The successful transfection of plasmids and expression the PLK1 kinase region and the PBD region in A549/DDP cells were demonstrated by the green fluorescence of EGFP-tagged Flag-KD and Flag-PBD (Fig. 4D). The binding of **B4** to the different regions of PLK1 in A549/DDP cells was assessed by immunoprecipitation-mass spectrometry (Fig. 4E). After incubating **B4** with A549/DDP cell extracts, the mixed solution was immunoprecipitated with anti-Flag antibody and analyzed by mass spectrometry for **B4** detection. **B4** was detected in Flag-PBD transfected sample (Fig. 4F, B). However, no **B4** was detected in Flag-KD transfected sample (Fig. 4F, C). These data collectively indicated that the small-molecule inhibitor **B4** directly binds to PLK1 PBD to inhibit its activity.

### B4 enhances cisplatin sensitivity in vitro and in vivo by blocking PLK1/PRC1 signaling

**Fig. 5:**
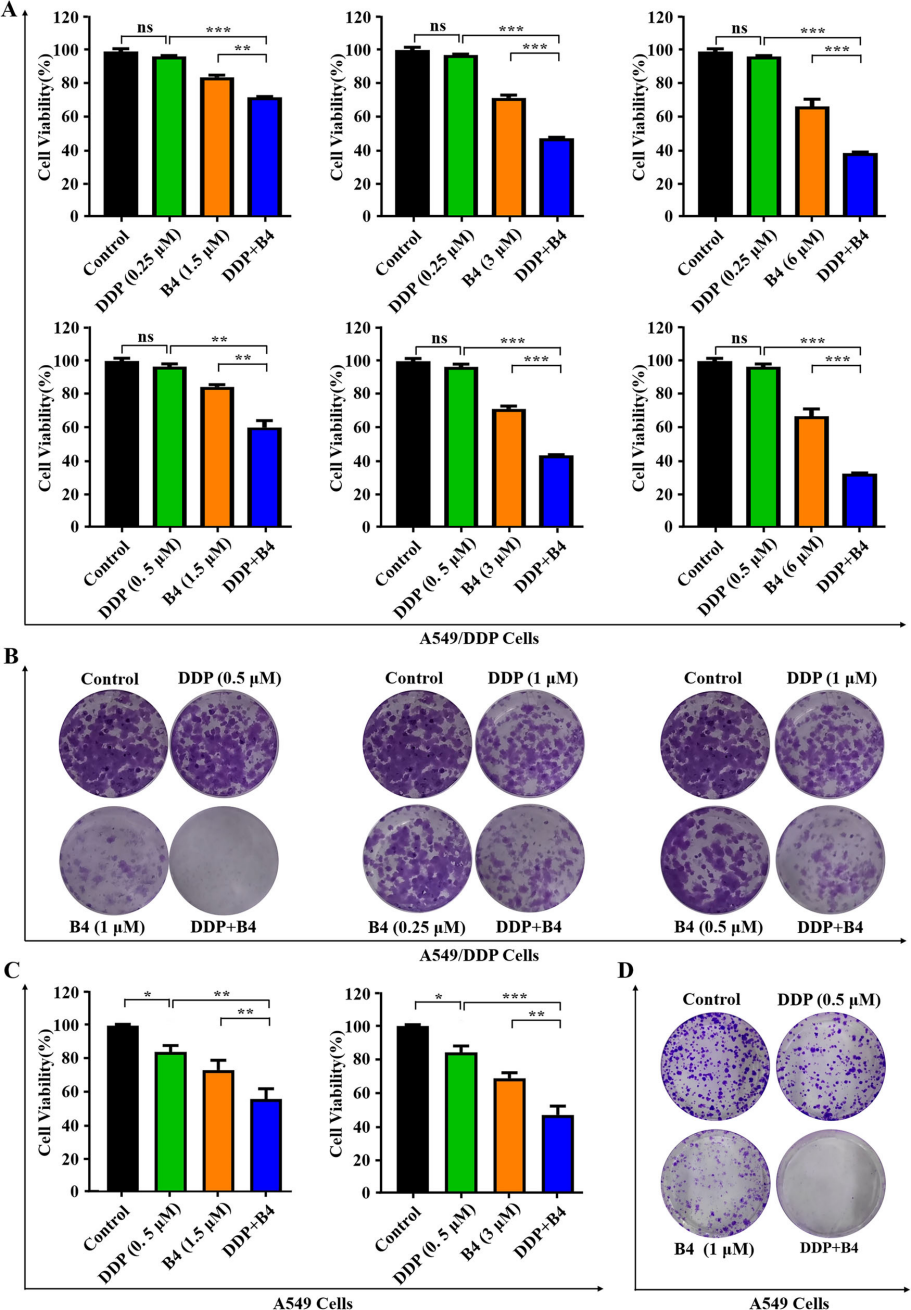
B4 combined with cisplatin improves chemoresistance in lung cancer in vitro. **A** Cell viability of A549/DDP cell lines treated with **B4** and cisplatin. Cell viability was determined on 48 h after treatment and proliferation index was calculated as fold change of cell viability. Columns, means (n = 3); bars, standard deviation (*p < 0.05, **p < 0.01, ***p < 0.001). **B** Colony formation assay in cells treated **B4**, cisplatin, or combination at 12 days. Images shown are representatives of three independent experiments. **C** Cell viability of A549 cell lines treated with **B4** and cisplatin. Cell viability was determined on 48 h after treatment and proliferation index was calculated as fold change of cell viability. Columns, means (n = 3); bars, standard deviation (*p < 0.05, **p < 0.01, ***p < 0.001). **D** Colony formation assay in cells treated **B4**, cisplatin, or combination at 12 days. Images shown are representatives of three independent experiments.

To evaluate the role of PLK1/PRC1 in chemotherapy resistance, we next investigated whether PLK1i could increase the cytotoxicity of cisplatin in A549/DDP. As shown in Fig. 5A, low dose cisplatin (0.25–0.5 μM) did not exhibit significant cytotoxicity in cisplatin resistant cells. However, **B4** enhanced the sensitivity of A549/DDP cells to cisplatin in a dose-dependent manner. We next investigated whether PLK1i had similar effect in colony formation of cisplatin resistant cells by treating with PLK1i and cisplatin. Consistent with results in cytotoxicity, the combination had a synergistic effect, which increased the sensitivity of A549/DDP cells to cisplatin for colony formation (Fig. 5B). However, the combination of **B4** and cisplatin enhanced the anti-tumor activity for cisplatin-sensitive A549 cells, which was additive and did not show a significant sensitization effect (Fig. 5C, D).

**Fig. 6:**
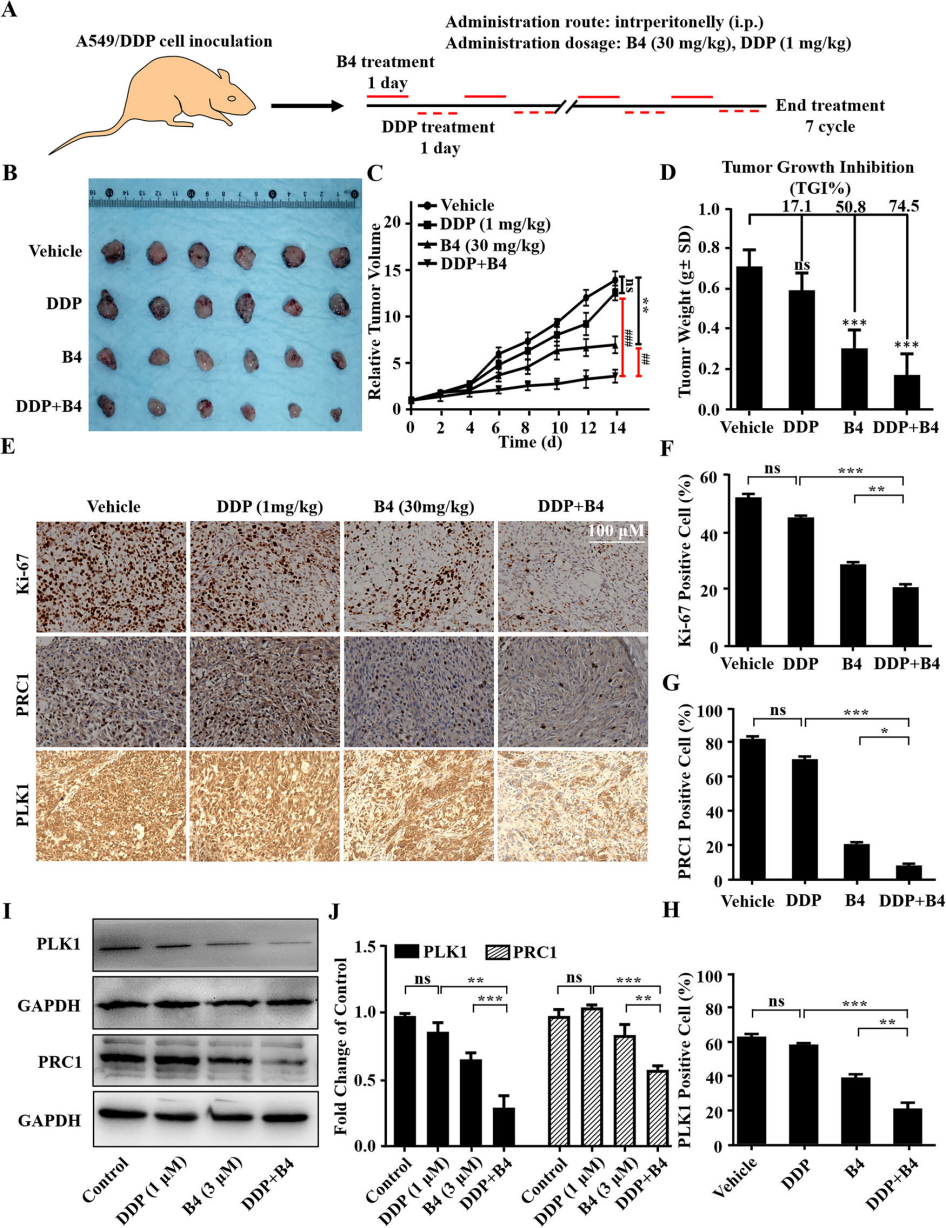
In vivo anticancer activity of B4 in an A549/DDP xenograft mice model. **A** Treatment scheme of **B4** in A549/DDP xenograft tumor model. **B** Images of excised tumor from different groups at time of euthanasia. **C** Relative tumor volume changes from different groups. Data are shown as the mean ± SD, n = 6, **p < 0.01, ^**##**^p < 0.01 and ^**###**^p < 0.001. **D** The average weight of excised tumors from different groups at time of euthanasia. Data are shown as the mean ± SD, n = 6, *p < 0.05, **p < 0.01 and ***p < 0.001. **E**–**H** **B4** inhibited Ki-67, PRC1 and PLK1 expression in tumor tissues. Tumors were excised at the end of treatment and then analyzed by immunohistochemistry (**E**). Three random fields of each sample were counted for the quantification of Ki-67 (**F**), PRC1 (**G**) and PLK1 (**H**) positive cells. Statistical analysis was carried out by GrapPad Prism with one-way ANOVA. **I**, **J** Expression of PLK1 and PRC1 in A549/DDP cells after **B4** treatment. Whole cell proteins were detected by western blotting (**I**) using indicated antibodies and quantified with imageJ (**J**). Data are presented as the mean ± SD. *p < 0.05, **p < 0.01 and ***p < 0.001.

To further confirm the effectiveness of combination therapy, we examined the anti-tumor effect of the combination of PLK1i and cisplatin in cisplatin-resistant and cisplatin-sensitive xenograft models, respectively. When the volume of tumors reached approximately 100 mm^3^, the mice were randomly divided into four groups and dosed intraperitoneally (i.p.) with the drug, **B4** (30 mg·kg^-1^ every two days), cisplatin (DDP, 1 mg·kg^-1^ every two days), and the combination of **B4** and cisplatin (every one day) (Figs. 6A and S5A). As depicted in Figs. 6B, C and S5B, the combination of cisplatin and **B4** significantly inhibited the growth of A549/DDP and A549 tumors compared to individual treatment. Moreover, the tumor growth inhibition (TGI) of A549/DDP tumors in the cisplatin and **B4** combination treatment group reached 74% (Fig. 6D), indicating that cisplatin and **B4** had a synergistic effect in inhibiting tumor growth. However, TGI of A549 tumors in the cisplatin and **B4** combination treatment group was only 53.6% (Fig. S5C), suggesting that PLKi could not enhance the sensitivity of cisplatin in A549 tumors. Furthermore, tumor tissues were also collected for analyzing the underlying anticancer mechanisms. As shown in Fig. 6E, F, the combination of **B4** with cisplatin significantly inhibited the proliferation of Ki-67-positive cells in drug-resistant lung cancer compared to the single drug-administration group. Taken together, these data indicated that **B4** enhanced sensitivity of the cisplatin-resistant tumors to cisplatin.

The levels of PRC1 and PLK1 in tumor cells were detected by IHC to explore whether the sensitization effect of **B4** for cisplatin was due to the inhibition of PLK1/PRC1 signaling pathway. PRC1 was detected in 25% of the **B4**-treated A549/DDP tumor tissues, while only 10% of the **B4** and cisplatin treated tumor tissues was PRC1 positive (Fig. 6E, G). PLK1 was detected in 38% of **B4**-treated A549/DDP tumor tissues, while this value decreased to 20% in tumor tissues treated with **B4** and cisplatin (Fig. 6E, H). In addition, we also analyzed PLK1 expression in A549 tumor models after the treatment of **B4** and cisplatin by IHC. As shown in Fig. S5D and S5E, both **B4** and combination therapy groups effectively reduced the positivity rate of PLK1 in tumor tissues, indicating that PLKi effectively regulated its targets in both drug-resistant and non-drug-resistant tumors.

To further confirm the above findings, we examined the expressions of PLK1 and PRC1 by protein immunoblotting after the treatment with **B4** and **B4**/cisplatin in A549/DDP cells. As depicted in Fig. 6I, J, both PLK1 and PRC1 expressions were significantly reduced in A549/DDP cells after treatment with **B4** and cisplatin, suggesting that **B4** was indeed able to increase the sensitivity of chemotherapy-resistant tumors to cisplatin by inhibiting PLK1/PRC1 signaling.

**Fig. 7:**
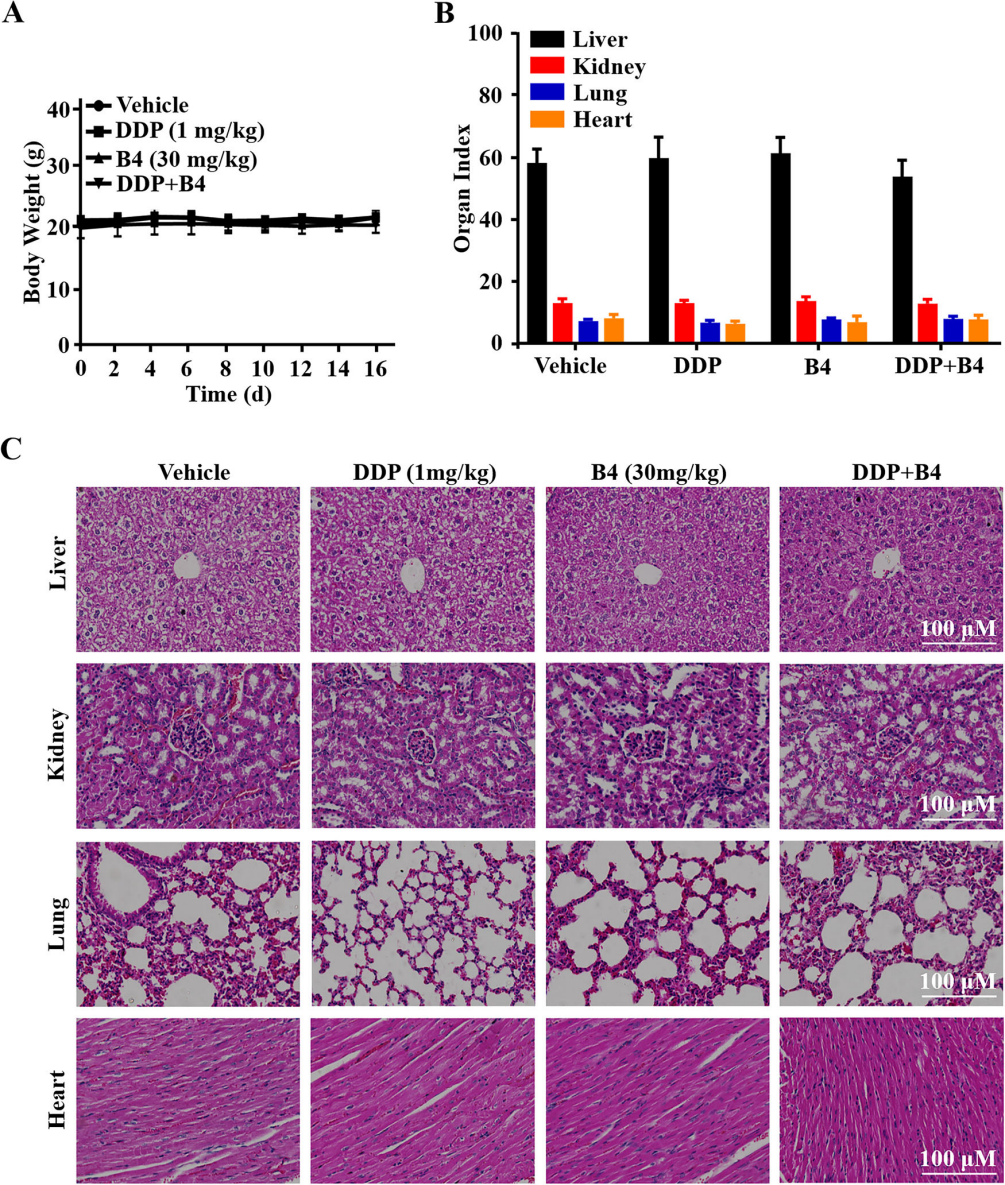
B4 has a safety profile in vivo. (**A**) Changes in body weights of the mice recorded over treatment period. Data are shown as the mean ± SD, (n = 6). (**B**) Weights of lung, hearts, livers, and kidneys from A549/DDP xenograft mice at the end of treament. Data are shown as the mean ± SD, n = 6, *p < 0.05. (**C**) H&E staining tissue images of the major organs obtain from each group mice in A549/DDP xenograft mice. Images shown are representatives of three independent experiments.

Lastly, the in vivo biosafety of **B4** was also evaluated by collecting, the body weight of mice and the weights of major organs during treatment. As shown in Fig. 7A, B, there were no significant changes in body and organ weights in the mice treated with **B4** as well as the combination of **B4** and cisplatin, compared with the control group. Moreover, histopathologic data also showed no significant damage to the major organs of mice (Fig. 7C). Thus, these data suggested that **B4** had a favorable biosafety profile in vivo.

### B4 inhibits PLK1/PRC1 signaling by disrupting the PRC1-PLK1 interaction

**Fig. 8:**
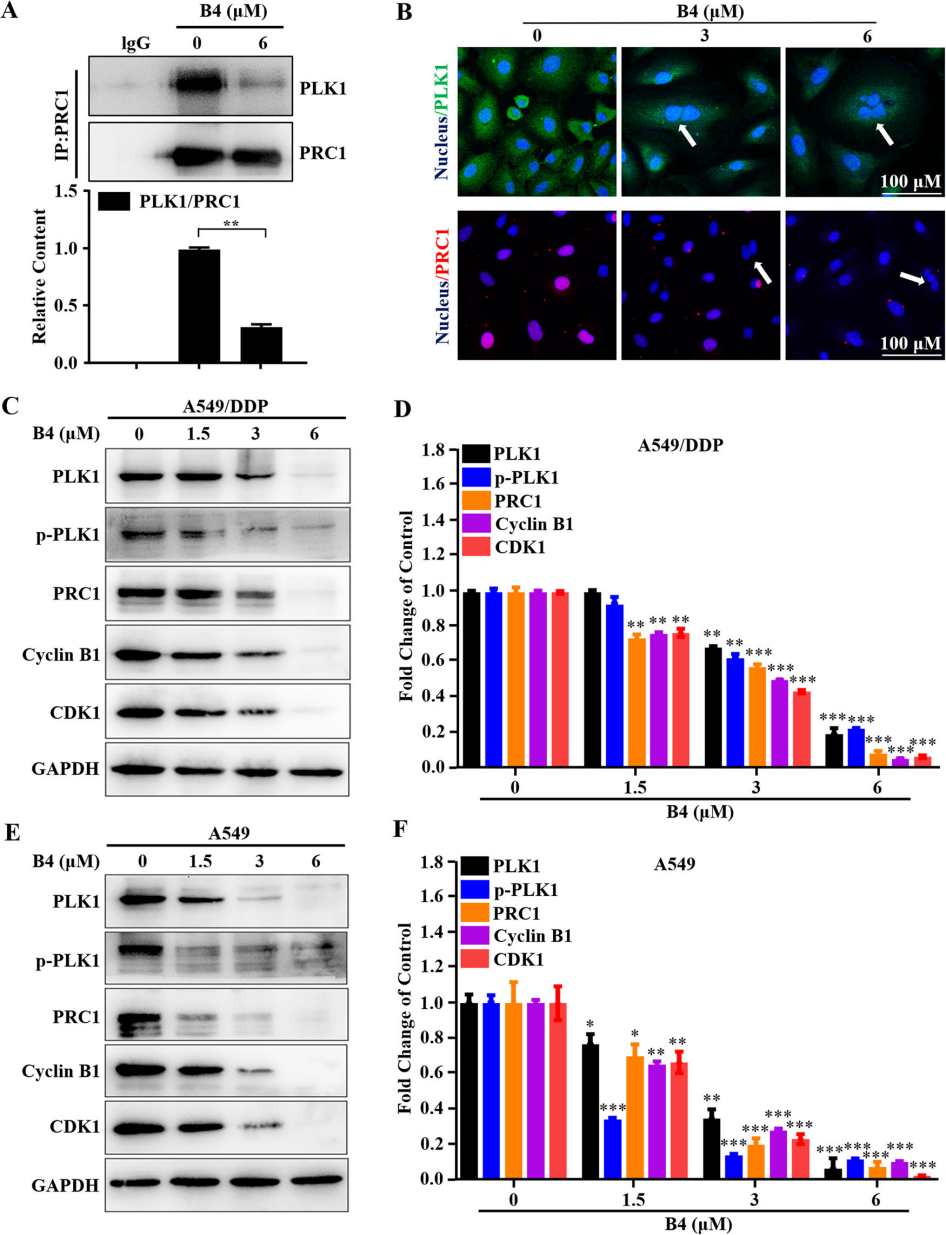
Mechanism study of B4 induced mitotic catastrophe. **A** Co-immunoprecipitation was conducted to examine the interaction between PLK1 and PRC1 in A549/DDP cells after **B4** treatment. **B** Immunostaining was performed to show the localization of PLK1 and PRC1 in A549/DDP cells after **B4** treatment at 48 h. Arrows indicate multinucleated cells. Images shown are representatives of three independent experiments. Expression of some cell cycle-related proteins after **B4** treatment in A549/DDP cells. Whole cell proteins were detected by western blotting (**C**) using indicated antibodies and quantified with imageJ (**D**). Each has the expression of GAPDH as the internal control. **E**, **F** Expression of some cell cycle-related proteins after **B4** treatment in A549 cells. Whole cell proteins were detected by western blotting (**E**) using indicated antibodies and quantified with imageJ (**F**). Data are presented as the mean ± SD. *p < 0.05, **p < 0.01 and ***p < 0.001.

Given that **B4** is a small molecule specifically targeting PLK1 PBD and the main function of PBD is to interact with phosphorylated docking proteins and recognize common sequences or docking sites, we investigated whether the inhibition of the PLK1/PRC1 signaling by **B4** was achieved by disrupting the interaction between PLK1 and PRC1 by coimmunoprecipitation. As shown in Fig. 8A, **B4** significantly reduced the amount of PLK1 captured by the PRC1 antibody. These results suggested that **B4** disrupted the interaction of PLK1 with PRC1. In addition, immunofluorescence imaging was conducted to observe the effect of **B4** on PLK1 and PRC1 in A549/DDP cells. In the presence of **B4**, multinucleated cells appeared during mitosis, suggesting that **B4** induced mitotic catastrophe in A549/DDP cells. Furthermore, the amount of PRC1 recruited by PLK1 was significantly reduced in anaphase in the presence of **B4** (Fig. 8B).

Since CDK1 can also phosphorylate PRC1 to drive cells into mitosis and cytokinesis [34**–**36], PLK1 inhibition by **B4** could be compensated by enhancing the function of CDK1. To verify whether **B4** affected the function of CDK1, we examined the protein abundance of the CDK1-Cyclin B1 complex in A549 and A549/DDP cells after **B4** treatment. As shown in Fig. 8C–F, PLK1 inhibition down-regulated the expression of the CDK1-Cyclin B1 complex.

## Discussion

In this study, we have identified a new small molecule **B4** capable of inhibiting spatiotemporal activity of PLK1 by disrupting the PLK1-PRC1 interaction. PLK1 is overexpressed in a variety of tumor cells, especially in drug-resistant cells, resulting in tumor progression and tumor resistance [32, 37]. The development of compounds such as **B4** may lead to mechanistically distinct antineoplastic drug. We demonstrated a strategy for development this compound and established the efficacy of **B4** in disrupting the PLK1-PRC1 interaction in vitro and in vivo. Direct binding of the compound to PLK1 was established using SPR, NMR as well as CoIP-MS, which further provides epitope mapping to show areas of **B4** that specially bind to PLK1 PBD region.

The therapeutic potential of PLK1 inhibitors is determined by their binding site and affinity with the target [38]. SPR data showed that **B4** bound directly to PLK1 with an estimated KD value of 5.6 μM. Notably, **B4** specifically bound with the PBD region of PLK1, which was confirmed by molecular docking and Co-IP-MS data. **B4** disrupted the PLK1-PRC1 interaction and led to PLK1 KD inhibition-like mitotic catastrophes, suggesting that the compound could be used as a selective PLK1 inhibitor for cancer therapy.

PLK1 overexpression is thought to be associated with chemotherapy resistance, and inhibition of PLK1 may improve the sensitivity of lung cancer cells to chemotherapeutic agents. Our in vitro results indicated that **B4** enhanced the proliferation inhibitory effect of cisplatin on A549/DDP cells. Furthermore, **B4** combined with cisplatin more significantly inhibited tumor cell growth in a drug-resistant tumor model (A549/DDP).

In conclusion, we identified a PLK1 inhibitor **B4** and demonstrated an alternative strategy to suppress spatiotemporal activity of PLK1 by disrupting the PLK1-PRC1 interaction. Our data demonstrate that **B4** treatment was efficacious and well tolerated in mice by improving chemosensitivity of tumor cells to chemotherapy. The development of **B4**-like PLK1 inhibitors has the potential to address the unmet medical need in cancer treatment.

## Supplementary information

Supporting Information of Pharmacological inhibition of PLK1/PRC1 triggers mitotic catastrophe and sensitizes lung cancers to chemotherapy

## Data Availability

The data supporting the findings of this study are available from the corresponding author upon reasonable request.

## References

[1] Vasan N, Baselga J, Hyman DM. A view on drug resistance in cancer. Nature. 2019;575:299–309. 31723286 10.1038/s41586-019-1730-1 PMC8008476

[2] Marine JC, Dawson SJ, Dawson MA. Non-genetic mechanisms of therapeutic resistance in cancer. Nat Rev Cancer. 2020;20:743–56. 33033407 10.1038/s41568-020-00302-4

[3] Boumahdi S, de Sauvage FG. The great escape: tumour cell plasticity in resistance to targeted therapy. Nat Rev Drug Discov. 2020;19:39–56. 31601994 10.1038/s41573-019-0044-1

[4] Ward RA, Fawell S, Floc’h N, Flemington V, McKerrecher D, Smith PD. Challenges and opportunities in cancer drug resistance. Chem Rev. 2021;121:3297–351. 32692162 10.1021/acs.chemrev.0c00383

[5] Robey RW, Pluchino KM, Hall MD, Fojo AT, Bates SE, Gottesman MM. Revisiting the role of ABC transporters in multidrug-resistant cancer. Nat Rev Cancer. 2018;18:452–64. 29643473 10.1038/s41568-018-0005-8 PMC6622180

[6] Redmond KM, Wilson TR, Johnston PG, Longley DB. Resistance mechanisms to cancer chemotherapy. Front Biosci. 2008;13:5138–54. 18508576 10.2741/3070

[7] Roesch A. Tumor heterogeneity and plasticity as elusive drivers for resistance to MAPK pathway inhibition in melanoma. Oncogene. 2015;34:2951–7. 25109330 10.1038/onc.2014.249

[8] Hata AN, Niederst MJ, Archibald HL, Caraballo MG, Siddiqui FM, Mulvey HE, et al. Tumor cells can follow distinct evolutionary paths to become resistant to epidermal growth factor receptor inhibition. Nat Med. 2016;22:262–9. 26828195 10.1038/nm.4040 PMC4900892

[9] Fong CY, Gilan O, Lam EYN, Rubin AF, Ftouni S, Tyler D, et al. BET inhibitor resistance emerges from leukaemia stem cells. Nature. 2015;525:538–42. 26367796 10.1038/nature14888 PMC6069604

[10] Wu YL, Tsuboi M, He J, John T, Grohe C, Majem M, et al. Osimertinib in resected EGFR-mutated non-small-cell lung cancer. N Engl J Med. 2020;383:1711–23. 32955177 10.1056/NEJMoa2027071

[11] Wang Y, Yao M, Li C, Yang K, Qin X, Xu L, et al. Targeting ST8SIA6-AS1 counteracts KRASG^12C^ inhibitor resistance through abolishing the reciprocal activation of PLK1/c-Myc signaling. Exp Hematol Oncol. 2023;12:105. 38104151 10.1186/s40164-023-00466-3 PMC10724920

[12] Zheng D, Li J, Yan H, Zhang G, Li W, Chu E, et al. Emerging roles of Aurora-A kinase in cancer therapy resistance. Acta Pharm Sin B. 2023;13:2826–43. 37521867 10.1016/j.apsb.2023.03.013 PMC10372834

[13] Wang X, Veeraraghavan J, Liu CC, Cao XX, Qin L, Kim JA, et al. Therapeutic targeting of nemo-like kinase in primary and acquired endocrine-resistant breast cancer. Clin Cancer Res. 2021;27:2648–62. 33542078 10.1158/1078-0432.CCR-20-2961 PMC8653766

[14] Yu Z, Deng P, Chen Y, Liu S, Chen J, Yang Z, et al. Inhibition of the PLK1coupled cell cycle machinery overcomes resistance to oxaliplatin in colorectal cancer. Adv Sci. 2021;8:2100759. 10.1002/advs.202100759 PMC8655181 34881526

[15] Eckerdt F, Strebhardt K. Polo-like kinase 1: Target and regulator of anaphase-promoting complex/cyclosome-dependent proteolysis. Cancer Res. 2006;66:6895–8. 16849530 10.1158/0008-5472.CAN-06-0358

[16] Ciardo D, Haccard O, Narassimprakash H, Chiodelli V, Goldar A, Marheineke K. Polo-like kinase 1 (Plk1) is a positive regulator of DNA replication in the Xenopus in vitro system. Cell Cycle. 2020;19:1817–32. 32573322 10.1080/15384101.2020.1782589 PMC7469467

[17] Eckerdt F, Yuan J, Strebhardt K. Polo-like kinases and oncogenesis. Oncogene. 2005;24:267–76. 15640842 10.1038/sj.onc.1208273

[18] Weichert W, Schmidt M, Gekeler V, Denkert C, Stephan C, Jung K, et al. Polo-like kinase 1 is overexpressed in prostate cancer and linked to higher tumor grades. Prostate. 2004;60:240–5. 15176053 10.1002/pros.20050

[19] Wu MJ, Wang Y, Yang D, Gong Y, Rao F, Liu R, et al. A PLK1 kinase inhibitor enhances the chemosensitivity of cisplatin by inducing pyroptosis in oesophageal squamous cell carcinoma. EBioMedicine. 2019;4:1244–55. 10.1016/j.ebiom.2019.02.012 PMC6442225 30876762

[20] Montaudon E, Nikitorowicz-Buniak J, Sourd L, Morisset L, Botty RE, Huguet L, et al. PLK1 inhibition exhibits strong anti-tumoral activity in CCND1-driven breast cancer metastases with acquired palbociclib resistance. Nat Commun. 2020;11:4053. 32792481 10.1038/s41467-020-17697-1 PMC7426966

[21] Tan J, Li ZM, Lee PL, Guan PY, Aau MY, Lee ST, et al. PDK1 signaling toward PLK1-MYC activation confers oncogenic transformation, tumor-initiating cell activation, and resistance to mtor-targeted therapy. Cancer Discov. 2013;3:1156–71. 23887393 10.1158/2159-8290.CD-12-0595

[22] Strebhardt K, Ullrich A. Targeting polo-like kinase 1 for cancer therapy. Nat Rev Cancer. 2006;6:321–30. 16557283 10.1038/nrc1841

[23] Elia AE, Rellos P, Haire LF, Chao JW, Ivins FJ, Hoepker K, et al. The molecular basis for phosphodependent substrate targeting and regulation of Plks by the Polo-box domain. Cell. 2003;115:83–95. 14532005 10.1016/s0092-8674(03)00725-6

[24] Elia AE, Cantley LC, Yaffe MB. Proteomic screen finds pSer/pThr-binding domain localizing Plk1 to mitotic substrates. Science. 2003;299:1228–31. 12595692 10.1126/science.1079079

[25] Barr FA, Sillje HH, Nigg EA. Polo-like kinases and the orchestration of cell division. Nature Rev Mol Cell Biol. 2004;5:429–40. 15173822 10.1038/nrm1401

[26] Lowery DW, Mohammad DH, Elia AE, Yaffe MB. The Polo-box domain: a molecular integrator of mitotic kinase cascades and Polo-like kinase function. Cell Cycle. 2004;3:128–31. 14712072

[27] Neef R, Gruneberg U, Kopajtich R, Li XL, Nigg EA, Sillje H, et al. Choice of Plk1 docking partners during mitosis and cytokinesis is controlled by the activation state of Cdk1. Nat Cell Biol. 2007;9:436–44. 17351640 10.1038/ncb1557

[28] Lanczky A, Gyorffy B. Web-based survival analysis tool tailored for medical research (KMplot): Development and implementation. J Med Internet Res. 2021;23:e27633. 34309564 10.2196/27633 PMC8367126

[29] Yun SM, Moulaei T, Lim D, Bang JK, Park JE, Shenoy SR, et al. Structural and functional analyses of minimal phosphopeptides targeting the polo-box domain of polo-like kinase 1. Nat Struct Mol Biol. 2009;16:876–82. 19597481 10.1038/nsmb.1628 PMC2721907

[30] Wolf G, Elez R, Doermer A, Holtrich U, Ackermann H, Stutte HJ, et al. Prognostic significance of polo-like kinase (PLK) expression in non-small cell lung cancer. Oncogene. 1997;14:543–9. 9053852 10.1038/sj.onc.1200862

[31] Yamamoto Y, Matsuyama H, Kawauchi S, Matsumoto H, Nagao K, Ohmi C, et al. Overexpression of polo-like kinase 1 (PLK1) and chromosomal instability in bladder cancer. Oncology. 2006;70:231–7. 16837776 10.1159/000094416

[32] Iliaki S, Beyaert R, Afonina IS. Polo-like kinase 1 (PLK1) signaling in cancer and beyond. Biochem Pharm. 2021;193:114747. 34454931 10.1016/j.bcp.2021.114747

[33] Bai Z, Zhou Y, Peng Y, Ye X, Ma L. Perspectives and mechanisms for targeting mitotic catastrophe in cancer treatment. Biochim Biophys Acta Rev Cancer. 2023;1878:188965. 37625527 10.1016/j.bbcan.2023.188965

[34] Jiang W, Jimenez G, Wells NJ, Hope TJ, Wahl GM, Hunter T, et al. PRC1: a human mitotic spindle-associated CDK substrate protein required for cytokinesis. Mol Cell. 1998;2:877–85. 9885575 10.1016/s1097-2765(00)80302-0

[35] Kurasawa Y, Earnshaw WC, Mochizuki Y, Dohmae N, Todokoro K. Essential roles of KIF4 and its binding partner PRC1 in organized central spindle midzone formation. EMBO J. 2004;23:3237–48. 15297875 10.1038/sj.emboj.7600347 PMC514520

[36] Mollinari C, Kleman JP, Jiang W, Schoehn G, Hunter T, Margolis RL. PRC1 is a microtubule binding and bundling protein essential to maintain the mitotic spindle midzone. J Cell Biol. 2002;157:1175–86. 12082078 10.1083/jcb.200111052 PMC2173564

[37] Gheghiani L, Wang L, Zhang YW, Moore XTR, Zhang JL, Smith SC, et al. PLK1 induces chromosomal instability and overrides cell-cycle checkpoints to drive tumorigenesis. Cancer Res. 2021;81:1293–307. 33376114 10.1158/0008-5472.CAN-20-1377 PMC8026515

[38] Zhou Y, Yan F, Huo X, Niu M. Discovery of a potent PLK1-PBD small-molecule inhibitor as an anticancer drug candidate through structure-based design. Molecules. 2019;24:4351. 31795214 10.3390/molecules24234351 PMC6930574

