# Supplementary material for: Pharmacological inhibition of PLK1/PRC1 triggers mitotic catastrophe and sensitizes lung cancers to chemotherapy
Source: Cell Death Dis. 2025 May 12;16(1):374. doi: 10.1038/s41419-025-07708-8 (PMC12069692; doi:10.1038/s41419-025-07708-8)
Supplement: Supplementary file 1 — Supporting Information of Pharmacological inhibition of PLK1/PRC1 triggers mitotic catastrophe and sensitizes lung cancers to chemotherapy [file 41419_2025_7708_MOESM1_ESM.docx]

**Supporting Information**

Pharmacological inhibition of PLK1/PRC1 triggers mitotic catastrophe and sensitizes lung cancers to chemotherapy

Pingping Li^1,4^, Yufei Zhao^1,4^, Minghan Lu^1,4^, Chengfei Chen^1,4^, Yongkun Li^1^, Lingling Wang^3^, Shulan Zeng^1^, Yan Peng^1^, Hong Liang^1^, Guohai Zhang^1,2✉^

^1^Key Laboratory for Chemistry and Molecular Engineering of Medicinal Resources (Ministry of Education of China), Collaborative Innovation Center for Guangxi Ethnic Medicine, Guangxi Key Laboratory of Chemistry and Molecular Engineering of Medicinal Resources, School of Chemistry and Pharmaceutical Sciences, Guangxi Normal University, Guilin 541004, China.

^2^Joint Medical Research Center of Guangxi Normal University & Guilin Hospital of Chinese Traditional and Western Medicine, Guilin 541004, China.

^3^School of Comprehensive Health Management, Xihua University, Chengdu, 610039, China.

^4^ Authors contributed equally.

✉Corresponding Author: Guohai Zhang

Tel. & Fax: 86-0773-2120958

E-mail: zgh1207@gxnu.edu.cn (G.H. Zhang)

**This PDF file includes:**

**1. Supplementary methods and materials**

**2. Supplementary figures and legends**

**3.** **Full and uncropped western blots**

**Supplementary methods and materials**

**Cell viability assay**

A549 and A549/DDP cell lines were inoculated in 96 well plates, respectively. After 24 h, cells were treated with various concentrations of **B4** (0, 1.25, 2.5, 5, 10, 20 μM) and cultured at 37^o^C for 48 h. Then, the cells were washed with PBS, fixed in 4% paraformaldehyde, and stained with 0.1% crystal violet staining solution for nucleia staining. After staining, 33% acetic acid (100 μL per well) was added to dissolved the crystal violet and then the optical absorbance at 570 nm was measured using a multi-well plate reader. The test values were normalized to the control groups.

**PLK1 kinase assay**

The inhibitory assay of PLK1 kinase was performed using a Dowex resin capture technique. In this assay, 85 μM αCasein (Sigma) was phosphorylated by 3 nM PLK1 in the presence of ATP (40 μM) traced with ^33^P-γ-ATP in kinase buffer for 60 min. By addition of an acidic suspension of Dowex resin (SIGMA) the unreacted ATP was captured and separated from the supernatant which contained the phosphorylated substrate: this was then transferred into a new plate for radioactivity counting. The kinase reactions were conducted in a total assay volume of 5 μL in a white 384-well plate. IC_50_ values were determined from dose–response curves by the four-parameter nonlinear regression curve fitting method using Graph Prism software.

**Analyses of cell cycle**

A549 and A549/DDP cells were treated with **B4** (0, 1.5, 3, 6, 9, 12, 15 μM) for the indicated times and then harvested and fixed in 70% ethanol. Fixed cells were stained with 100 μg/mL RNase A (Solarbio) and 1mg/ml Propidium Iodide (Solarbio, C0080). The stained cells were analyzed for DNA content using Becton Dickinson FACS Aria II flow cytometer (San Jose, CA) and ModFit LT software. Each data point in this assay represents the average of three independent measurements, with error bars indicating standard deviation.

**Colony formation assay**

Single-cell suspensions of A549/DDP cells were plated (50 cells per well) in 6-well plates, and the medium with **B4** (0.25, 0.5, 1 μM) and DDP (0.5, 1 μM) were refreshed every 3 days for 12 days of culture. Colonies were fixed in 4% paraformaldehyde, stained with 0.1% crystal violet staining solution for nucleia staining, and photographed.

**Western blotting**

After treatment with **B4** (0, 1.5, 3, 6 μM) for 48 h, A549/DDP cells were lysed by NP40 buffer containing 1% PMSF for 30 min, and then centrifuged at 13000 rpm for 10 min. Protein concentration of the supernatants was quantified using a BCA assay. Appropriate loading buffer was added into the lysates and heated for 5 min in boiling water. Samples were separated on polyacrylamide SDS gels, transferred to nitrocellulose membrane and probed with primary antibodies as indicated. The membrane was incubated with goat anti-rabbit lgG or goat anti-mouse lgG antibodies and developed by an enhanced chemiluminescence imaging analyzer (Tanon-5200, Shanghai. China).

**Immunofluorescence**

A549/DDP cells were treated with **B4** (0, 3, 6 μM) for 48h. After treatment with **B4**, A549 cells were washed with precooling PBS, fixed in 4% paraformaldehyde, perforated with 0.5% Triton X-100 and then subjected to primary antibody co-incubation (anti-α-tubulin, anti-PLK1 and anti-PRC1 antibody), overnight at 4°C. Then, secondary antibodies were incubated at room temperature for 2 h. Finally, morphological observations were performed.

**Histopathological section analysis**

The dissected tissue was fixed with 4% formaldehyde solution and processed routinely for paraffin embedding. Sections were cut at around 5 µm thickness and placed on glass slides for haematoxylin and eosin (H&E) staining and immunohistochemistry (IHC) analysis. For H&E staining, the heart, liver, kidney, lung tissue sections were stained with hematoxyl and eosin. For IHC analysis, the slides of tumors were boiling in IHC antigen repair buffer (pH 8.0-9.0) for 20 min for epitope retrieval. After quenching endogenous peroxidase with 3% H_2_O_2_ for 10 min, the sections were blocked with 5% BSA for 30 min. The slides were incubated with anti-Ki67 antibody overnight at 4℃, incubated with HRP-polymer anti-rabbit for 30 min and detected with DAB colorimetric kit, followed by counterstained with hematoxylin. Images were acquired by the Cytation 5 multifunctional imaging system. Results were quantified by ImageJ software.

**Supplementary figures and legends**

**
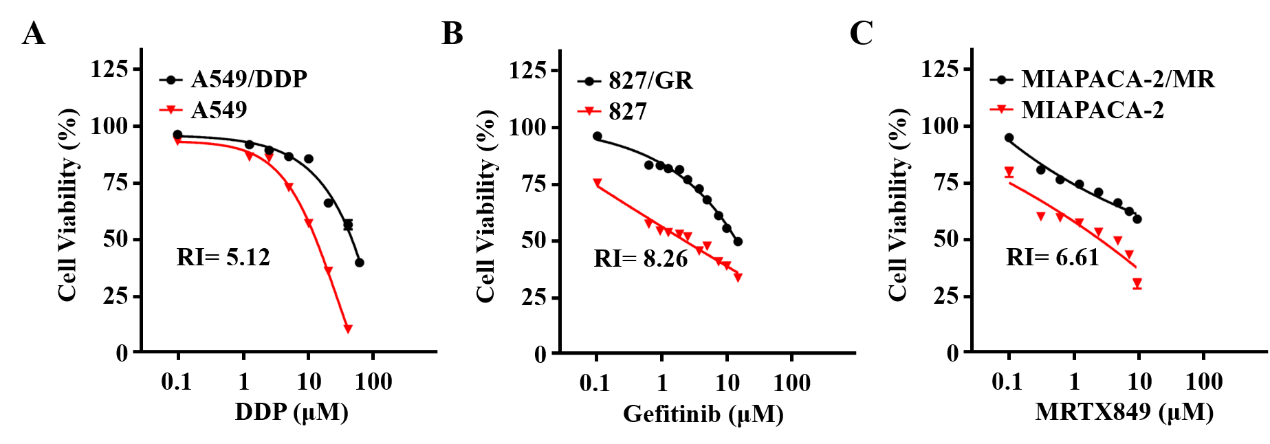
**

**Fig. S1** **Resistance index (RI) of parental cell lines and corresponding drug-resistant cell lines.** (A) Cell viability of DDP was tested on A549 cells and A549/DDP cells. (B) Cell viability of gefitinib was tested on HCC-827 cells and HCC-827/GR cells. (C) Cell viability of MRTX849 was tested on MIAPACA-2 cells and MIAPACA-2/MR cells. Data is expressed as mean ± SD, (n=3).

**
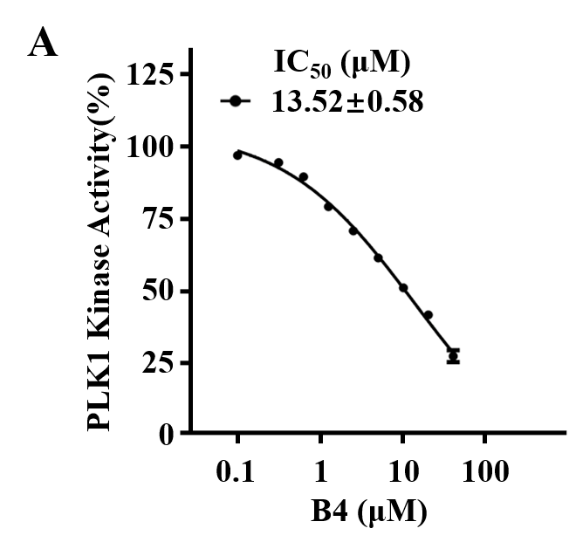
**

**Fig. S2** (A) Dose–response curves of **B4** on PLK1 kinase activity. The data are represented as mean ± SD of three independent experiments.

**
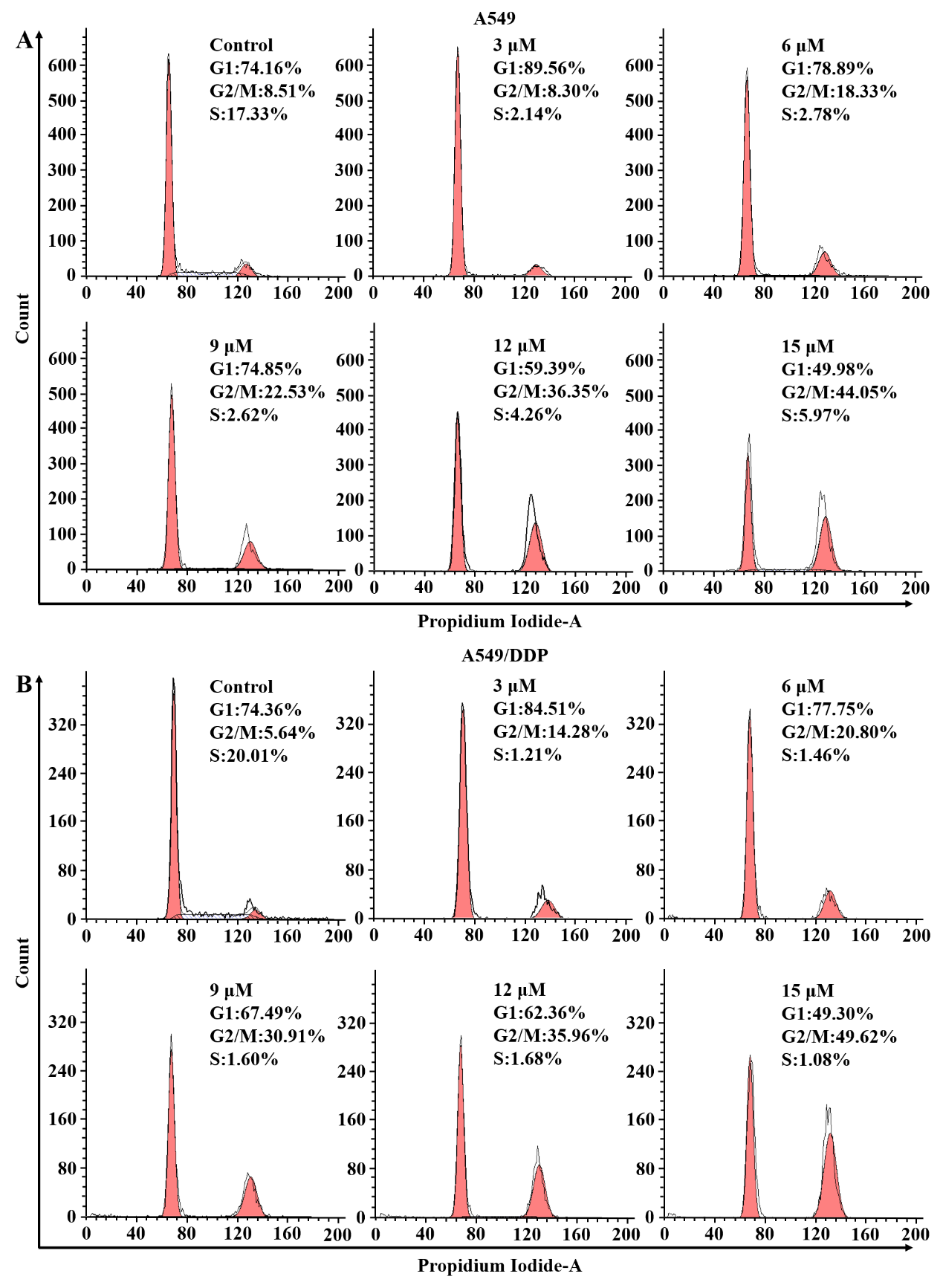
**

**Fig. S3** **Regulation of B4 on cell cycle progression.** (A, B) PI staining was used to investigate the effects of **B4** on the cell cycle of A549 cells (A) and A549/DDP cells (B). Data is processed through ModFit LT.


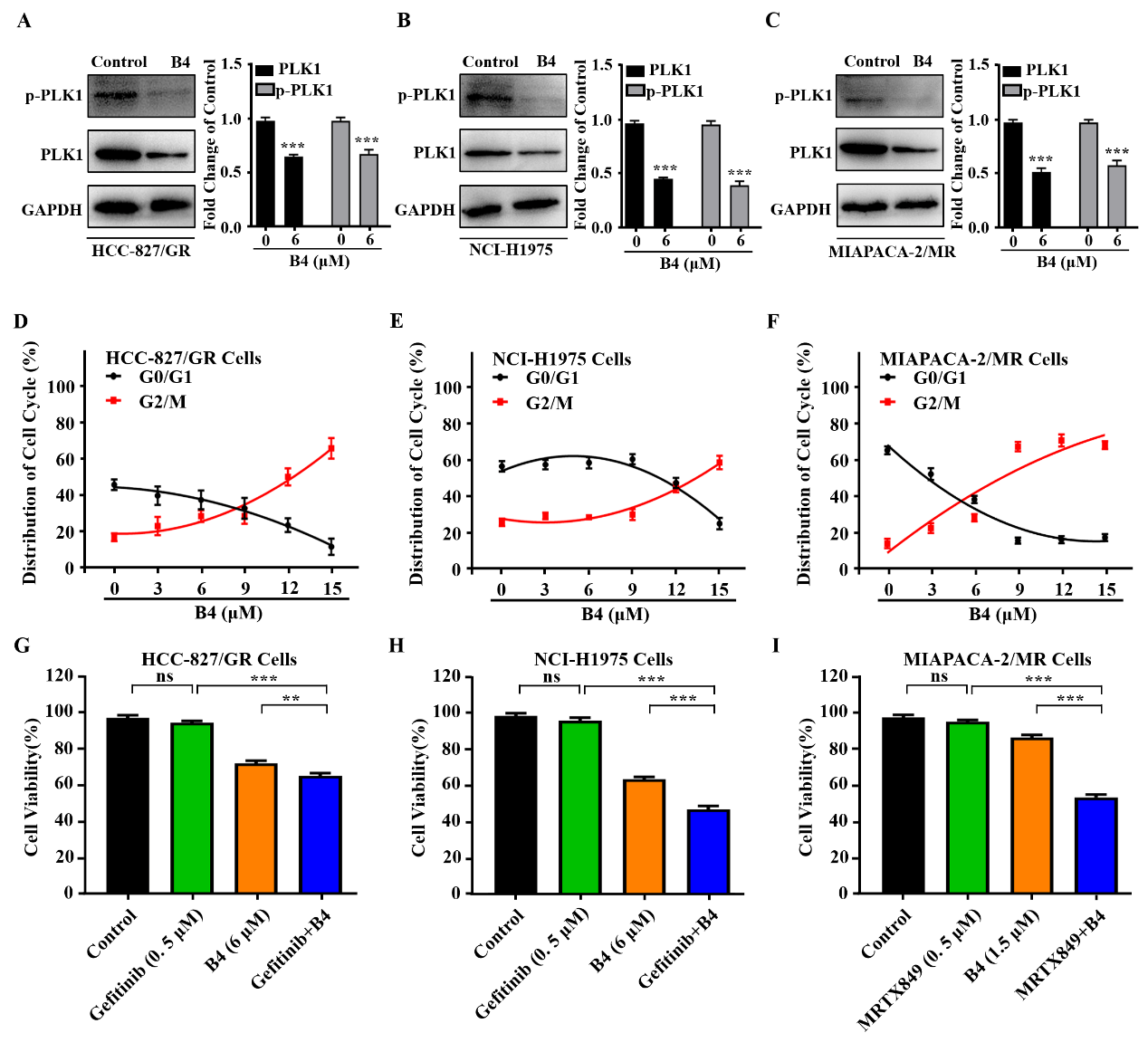


**Fig. S4** **Regulation of B4 on cell cycle progression.** (A-C) Protein abundance of PLK1 and p-PLK1 after **B4** treatment. Lysates from HCC-827/GR cells (A), NCI-H1975 cells (B) and MIAPACA-2/MR cells (C) after 48 h of **B4** treatment were detected by western blotting and quantified using imageJ. Data is expressed as mean ± SD, (n=3) **p < 0.01 and ***p < 0.001. (D-F) Effect of **B4** on cell cycle in HCC-827/GR cells (D), NCI-H1975 cells (E) and MIAPACA-2/MR cells (F). After treated with **B4** for 48 h, PI staining was used to examine the cell cycle of HCC-827/GR cells (D), NCI-H1975 cells (E) and MIAPACA-2/MR cells (F). Data is expressed as mean ± SD, (n=3). (G-I) Cell viability of HCC-827/GR cells (G), NCI-H1975 cells (H) and MIAPACA-2/MR cells (I) treated with **B4**+gefitinib and **B4**+MRTX849. Cell viability was determined on 48 h after treatment and proliferation index was calculated as fold change of cell viability. Error bars represent ± SD.


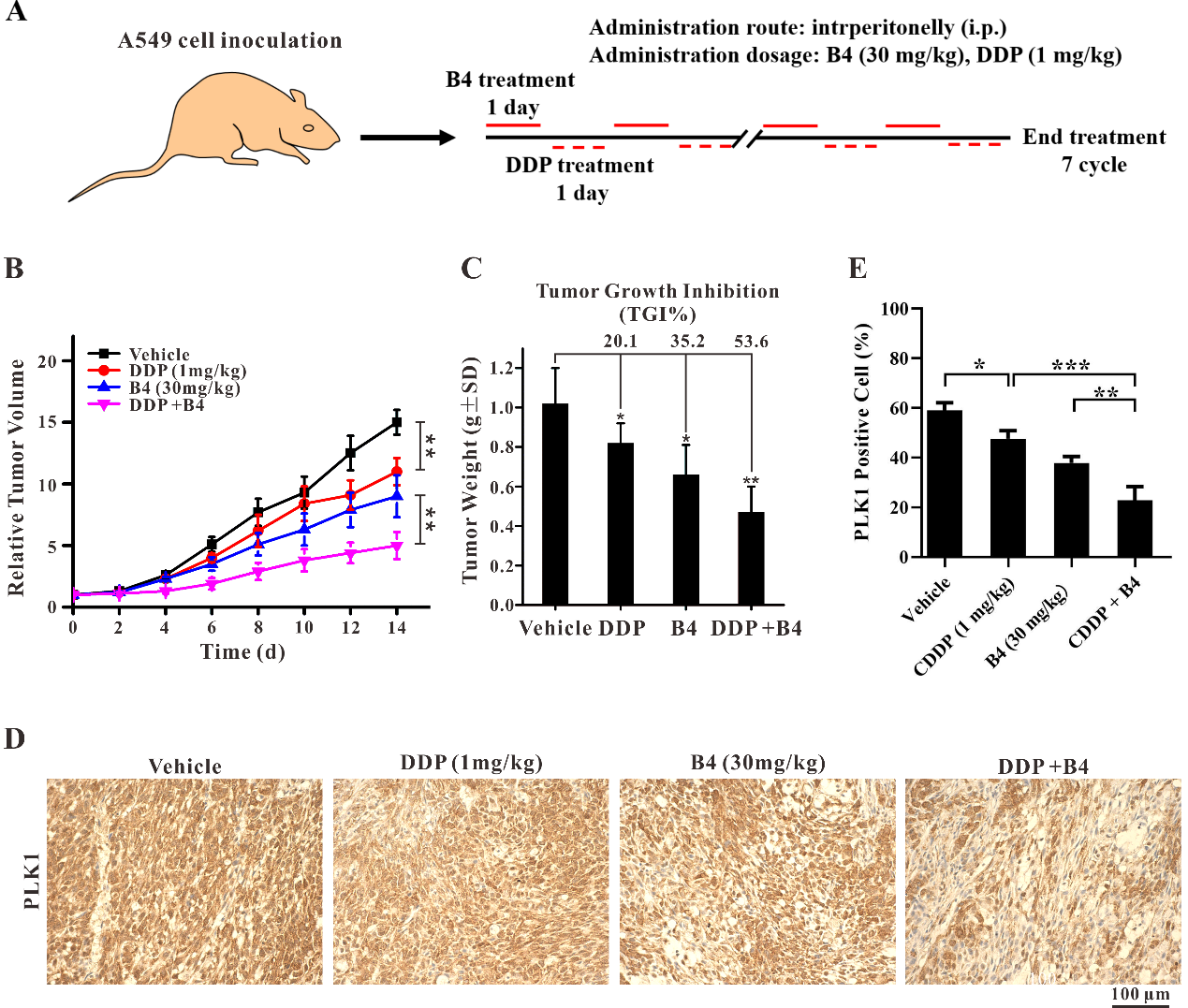


**Fig. S5** **In vivo anticancer activity of B4 in an A549 xenograft mice model.** (A) Treatment scheme of **B4** in A549 xenograft tumor model. (B) Relative tumor volume changes from different groups. Data are shown as the mean ± SD, n = 6, **p < 0.01 (C) The average weight of excised tumors from different groups at time of euthanasia. Data are shown as the mean ± SD, n = 6, *p < 0.05, **p < 0.01 and ***p < 0.001. (D, E) **B4** inhibited PLK1 expression in tumor tissues. Tumors were excised at the end of treatment and then analyzed by immunohistochemistry (D). Three random fields of each sample were counted for the quantification of PLK1 positive cells (E). Statistical analysis was carried out by GrapPad Prism with one-way ANOVA.

**Full and uncropped western blots**


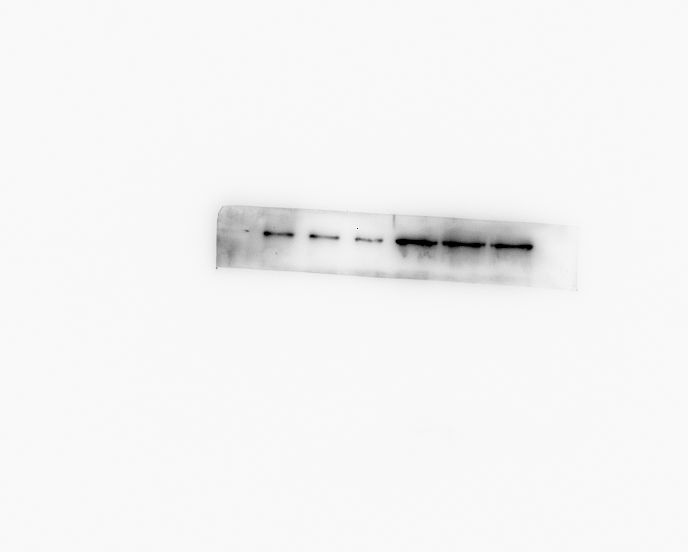

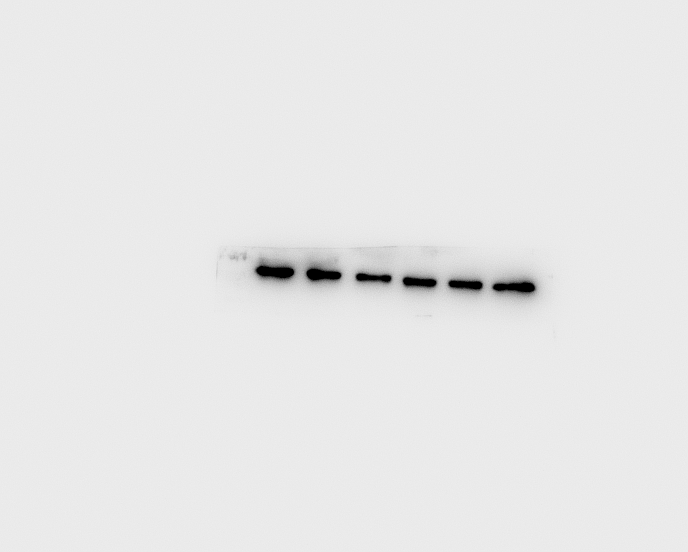


Western blot raw data in Fig. 2C


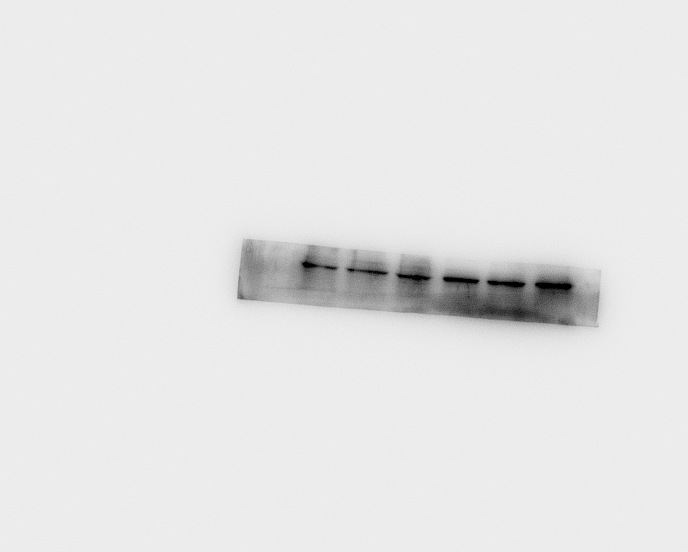

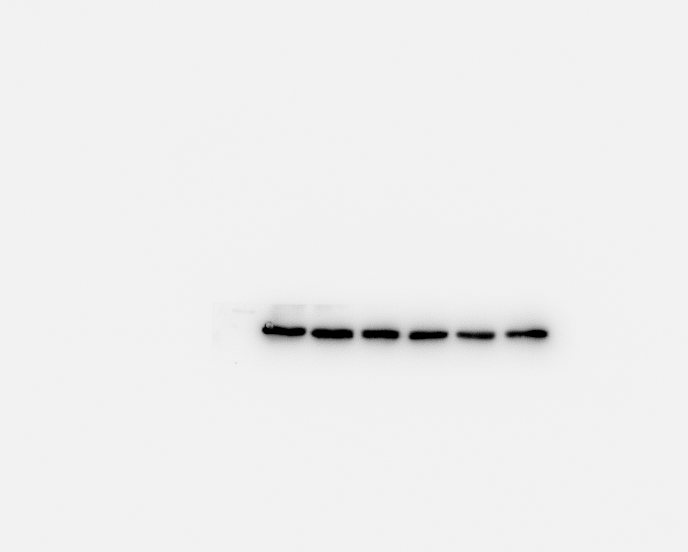


Western blot raw data in Fig. 2D


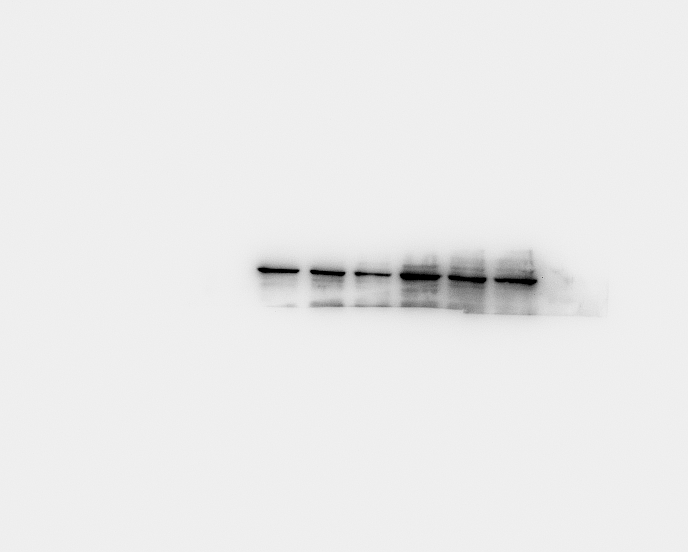

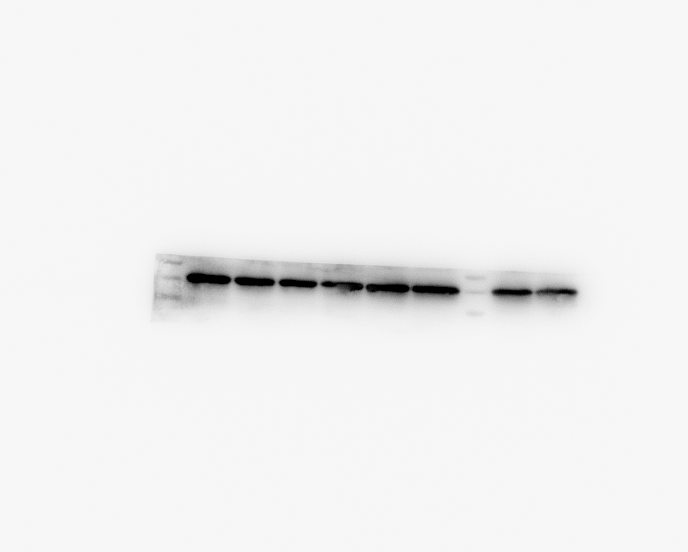


Western blot raw data in Fig. 2E


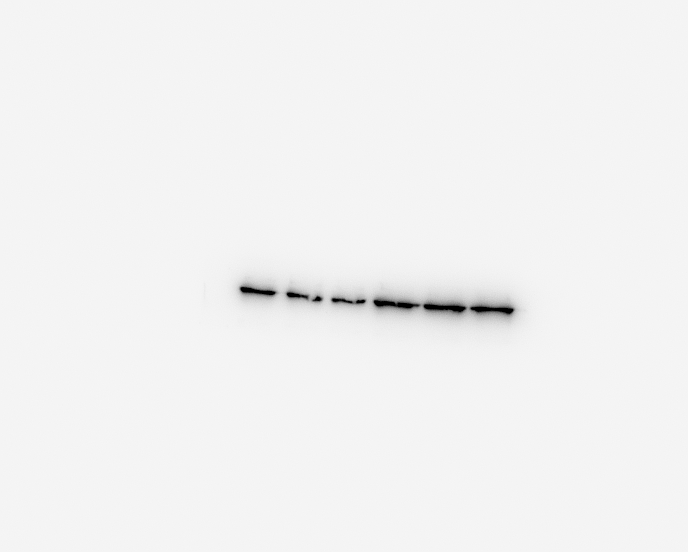

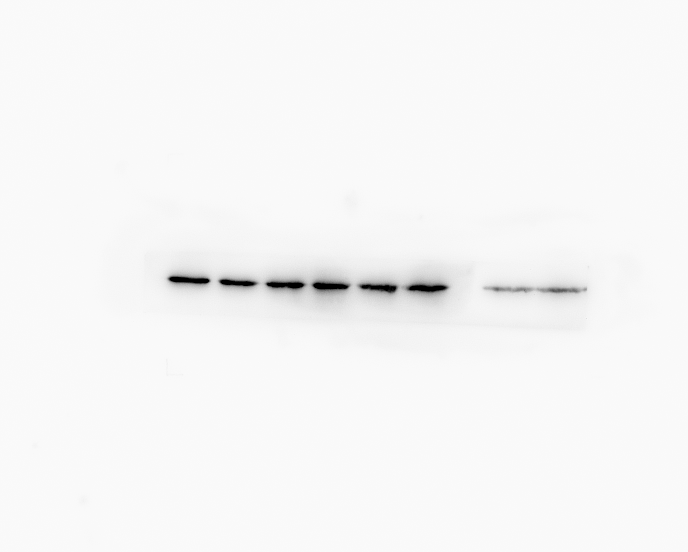


Western blot raw data in Fig. 2F


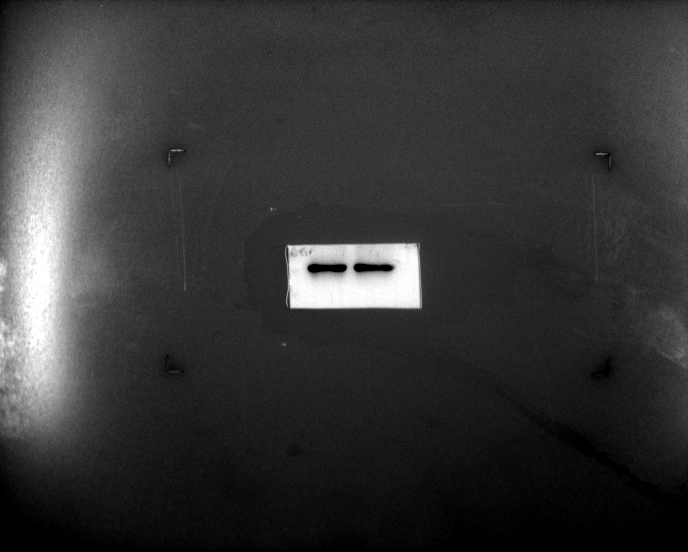

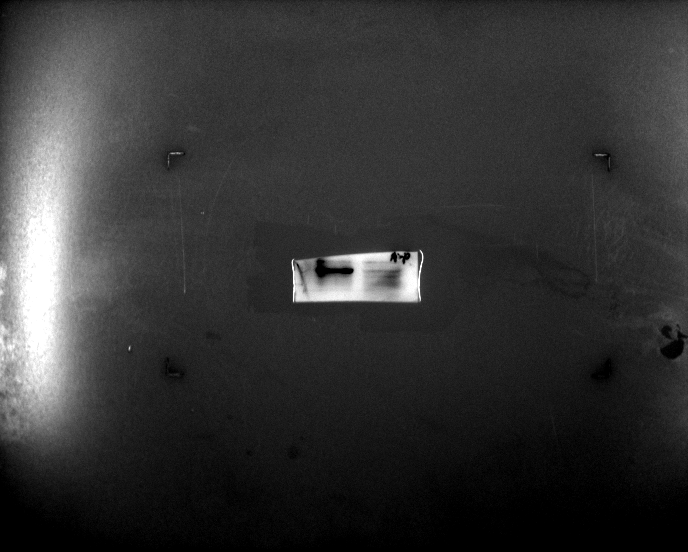

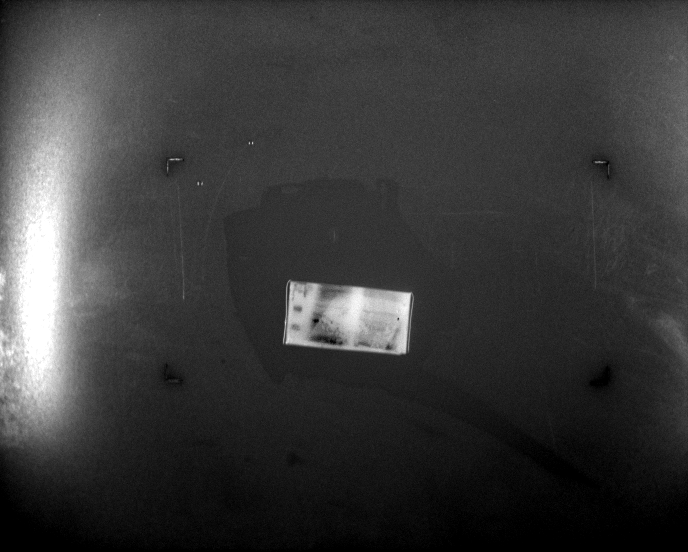


Western blot raw data in Fig. 3E


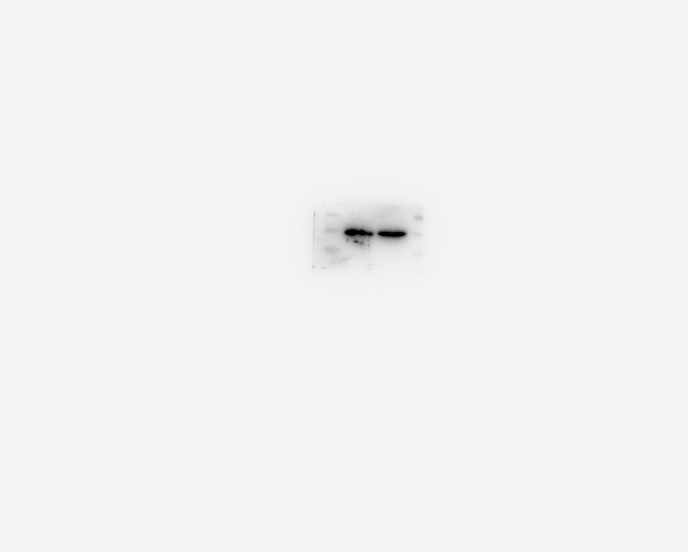

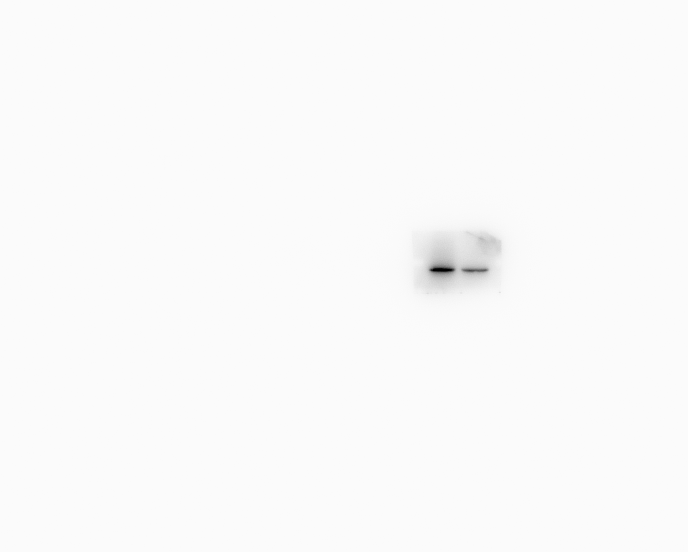

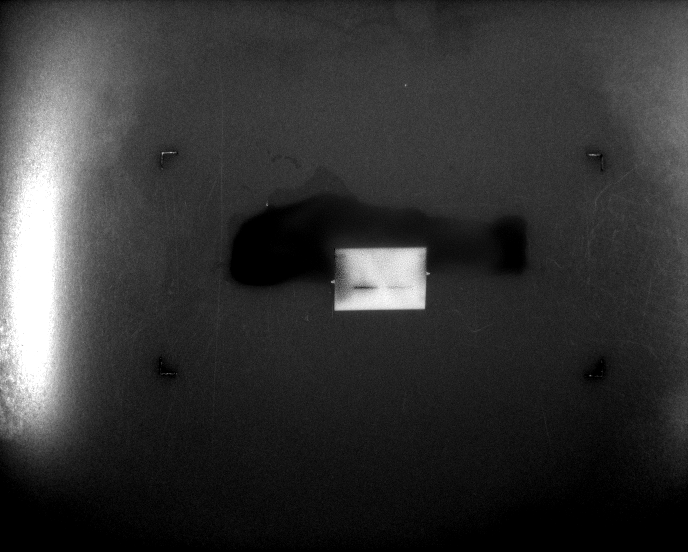


Western blot raw data in Fig. 3F


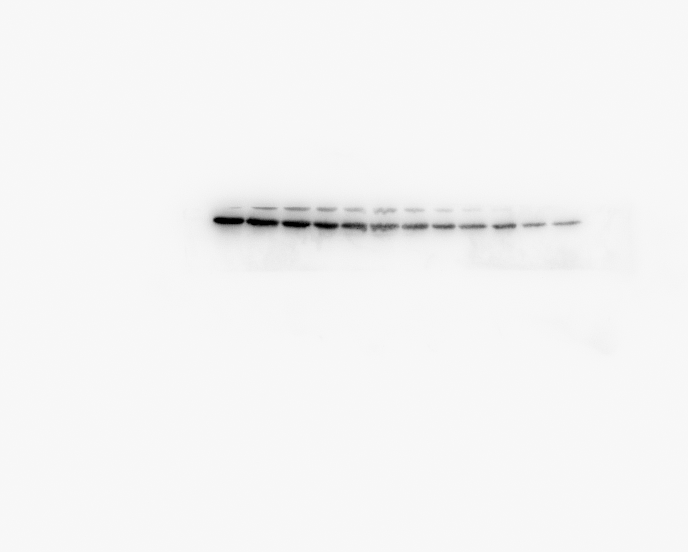

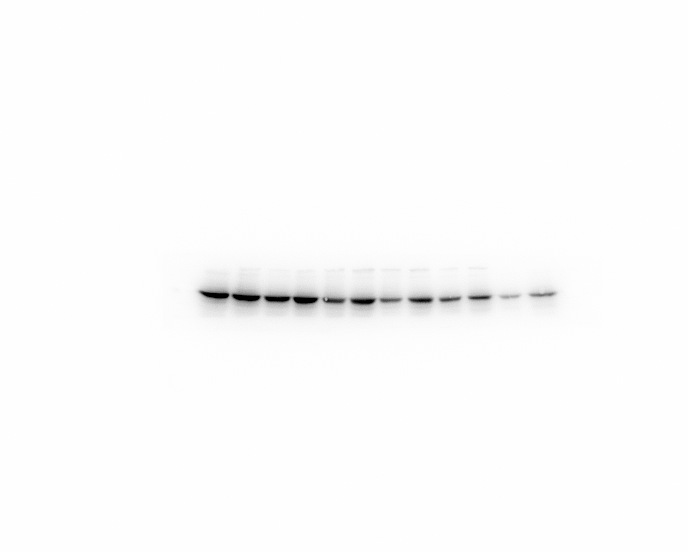

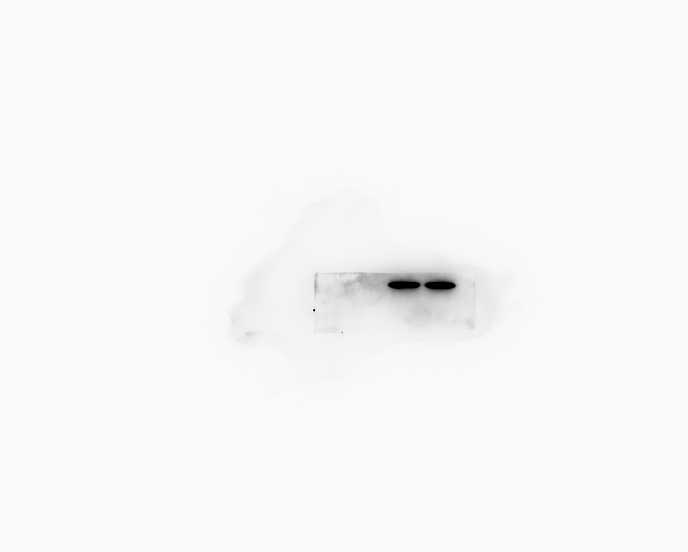

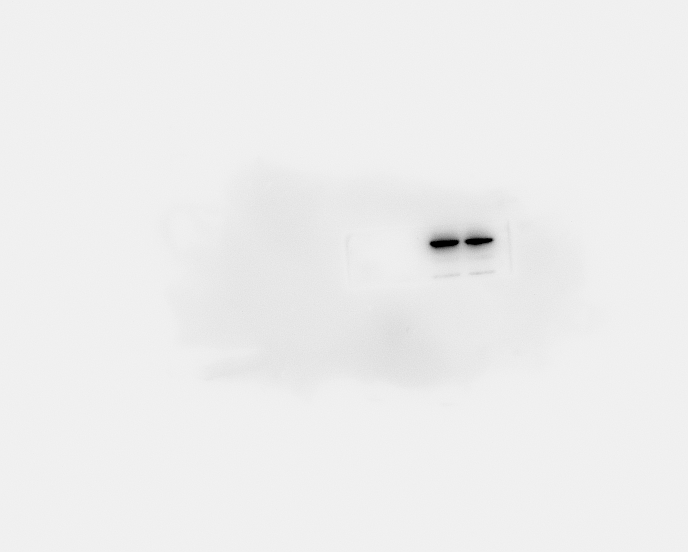


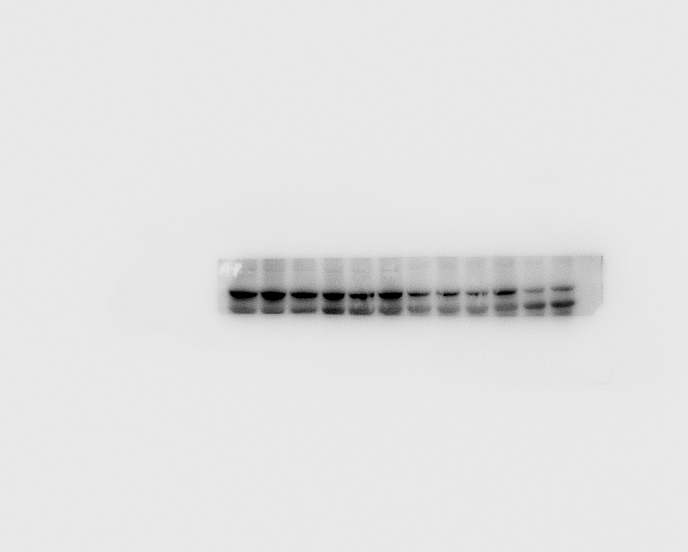

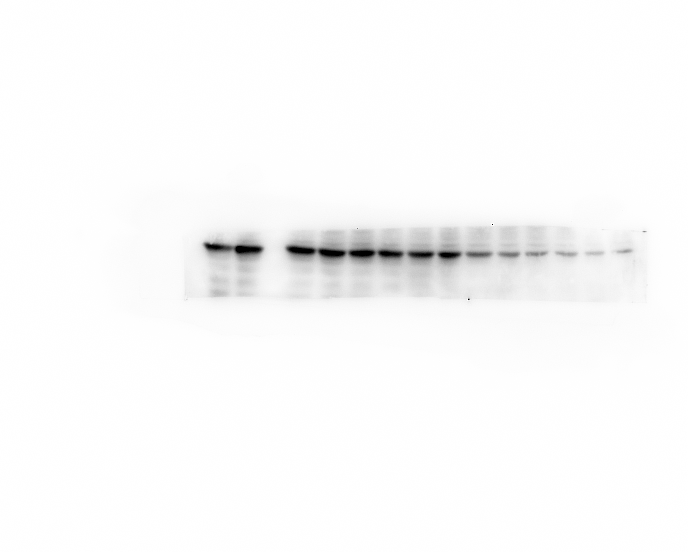

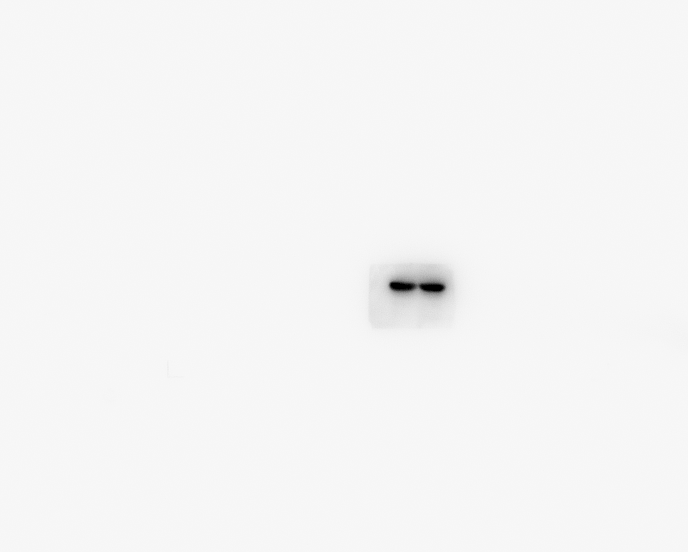

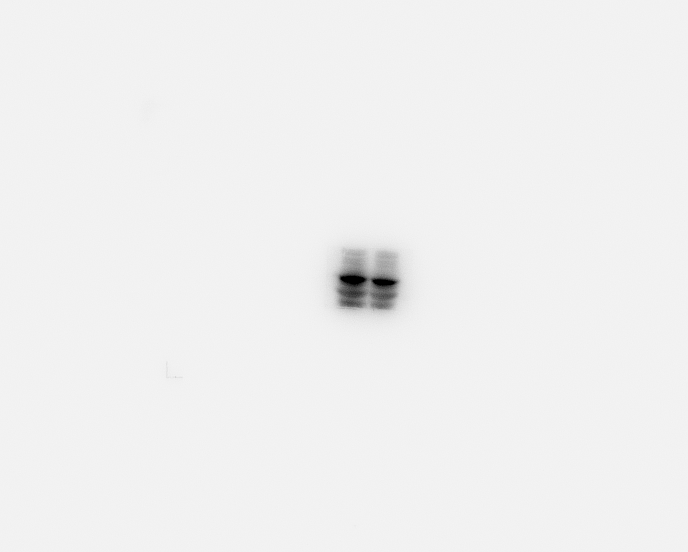


Western blot raw data in Fig. 4B


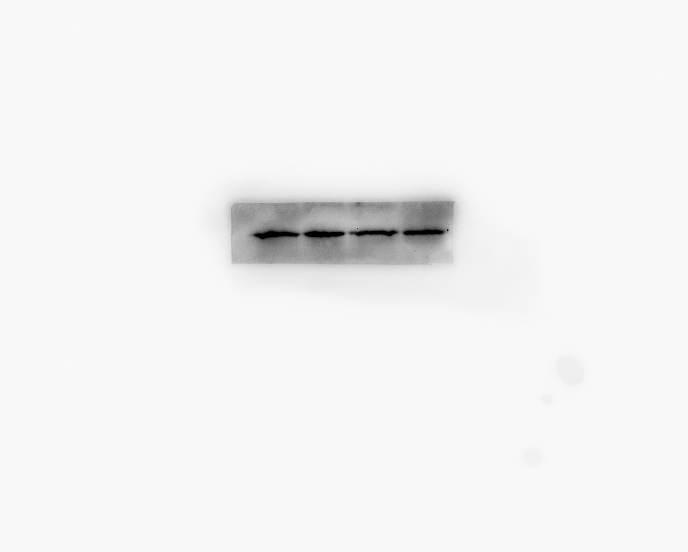

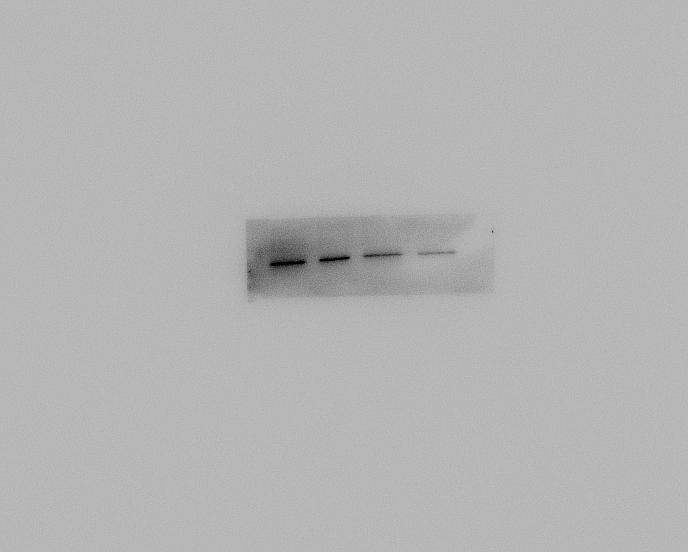

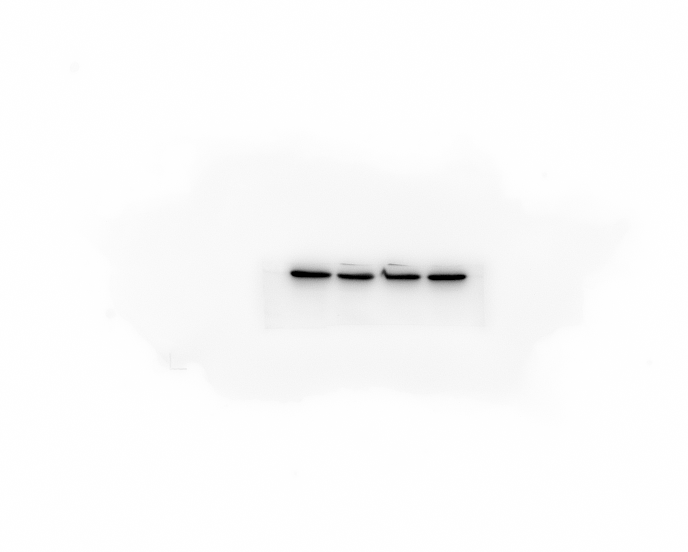

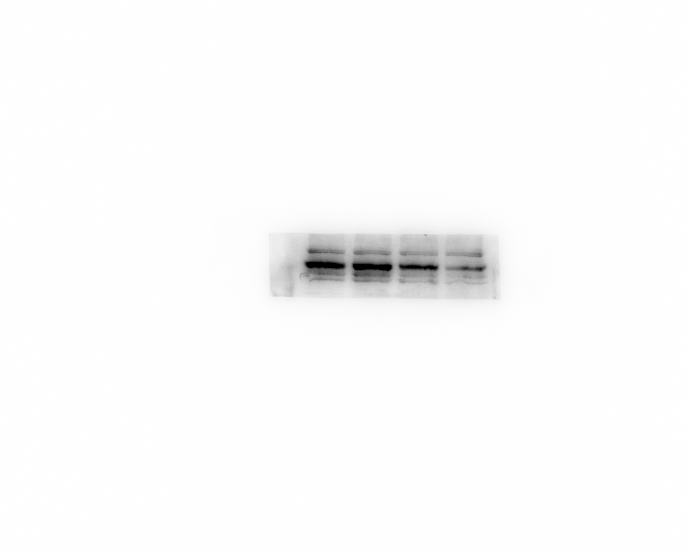


Western blot raw data in Fig. 6I


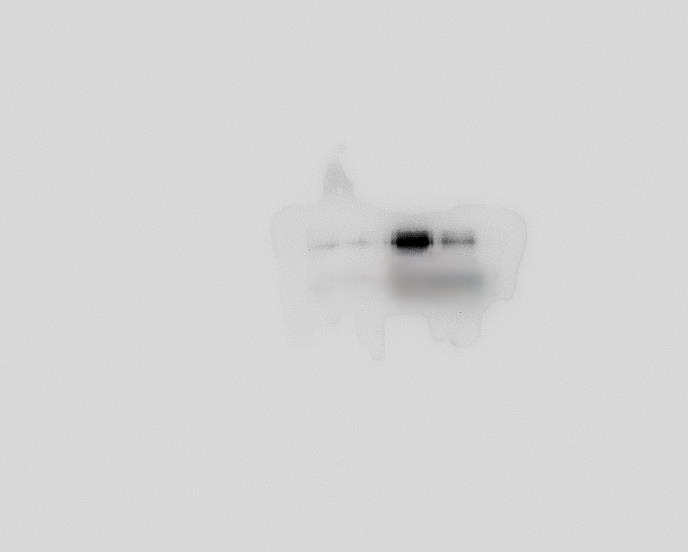

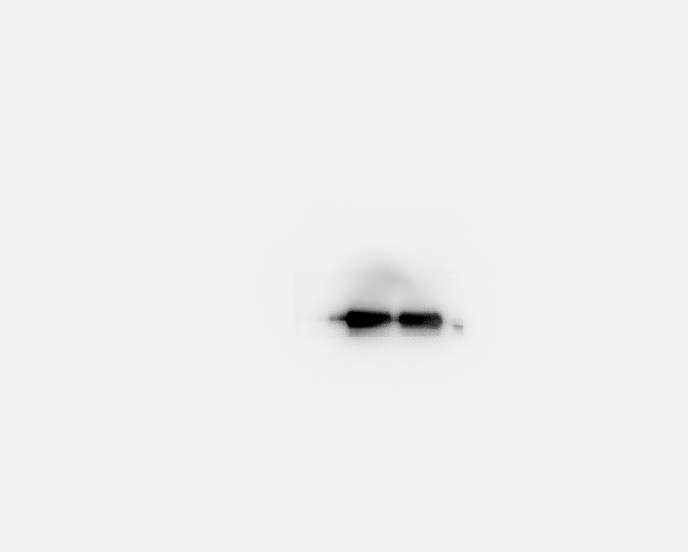


Western blot raw data in Fig. 8A


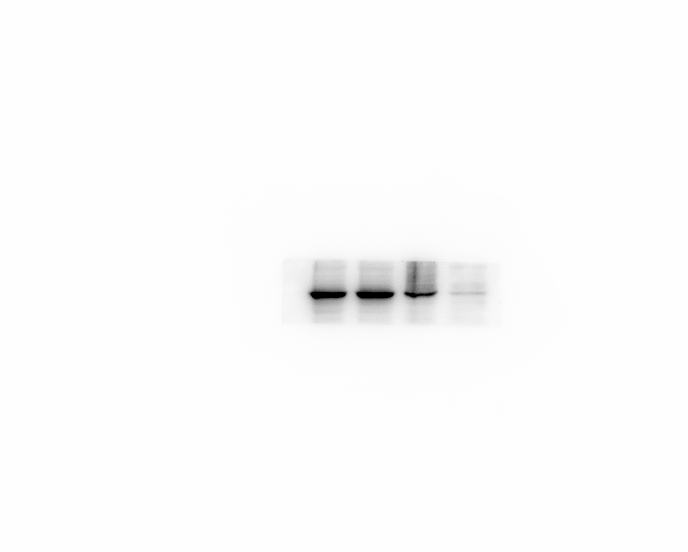

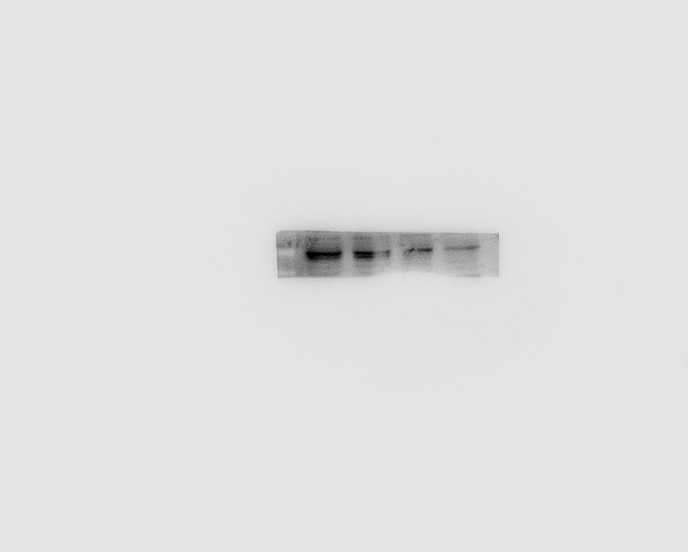

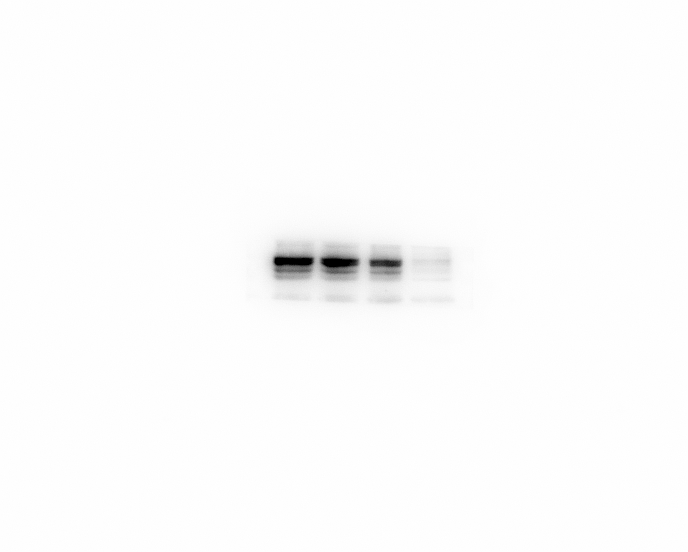

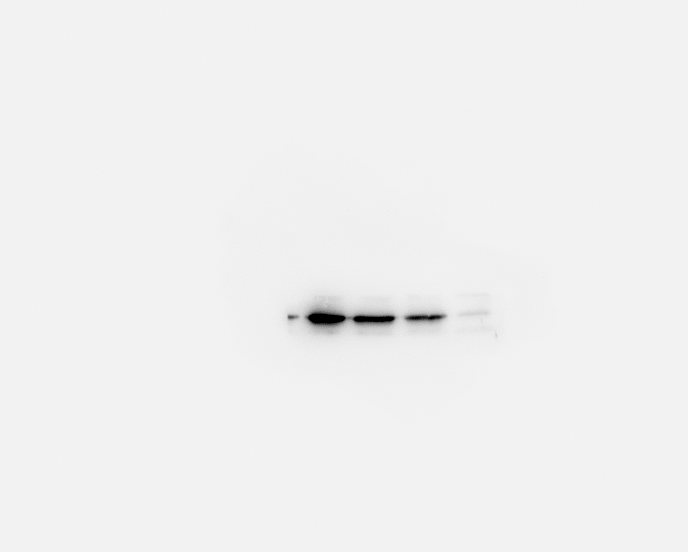

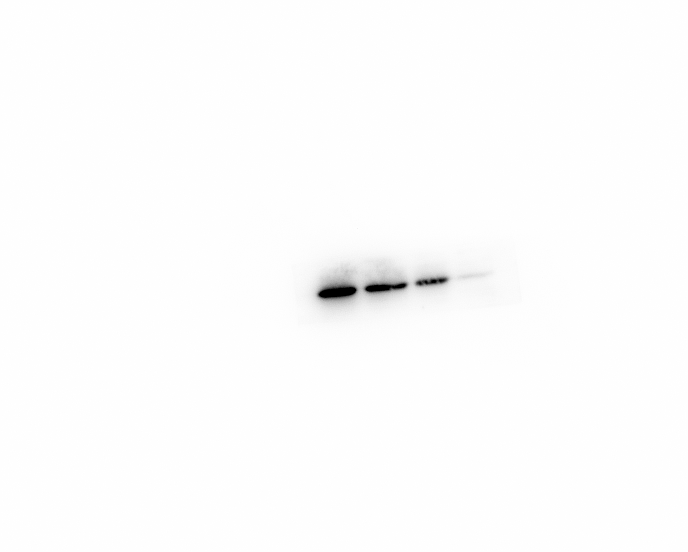

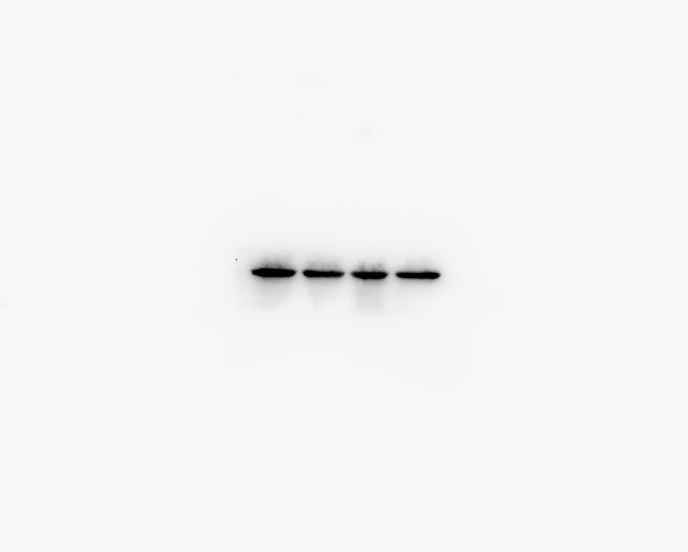


Western blot raw data in Fig. 8C


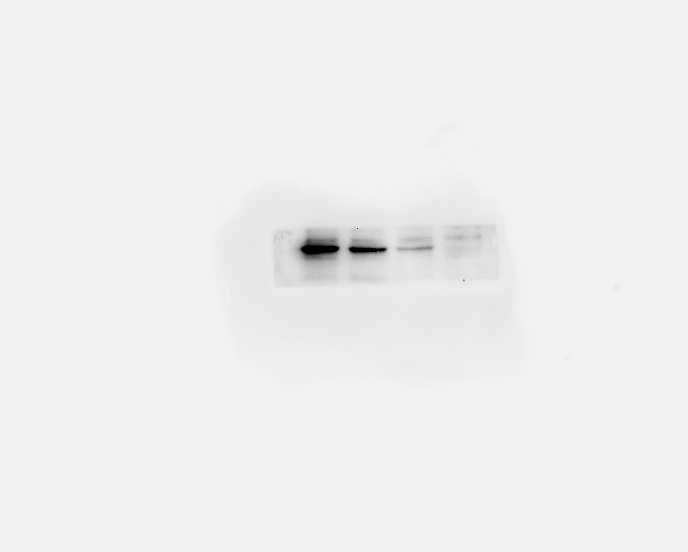

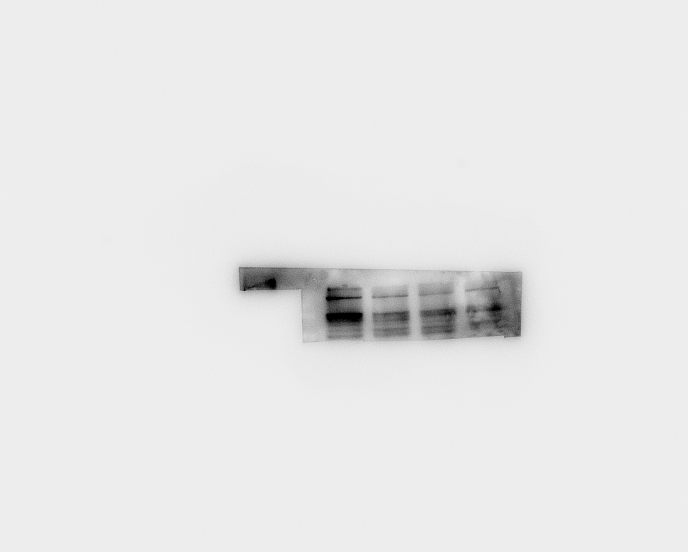

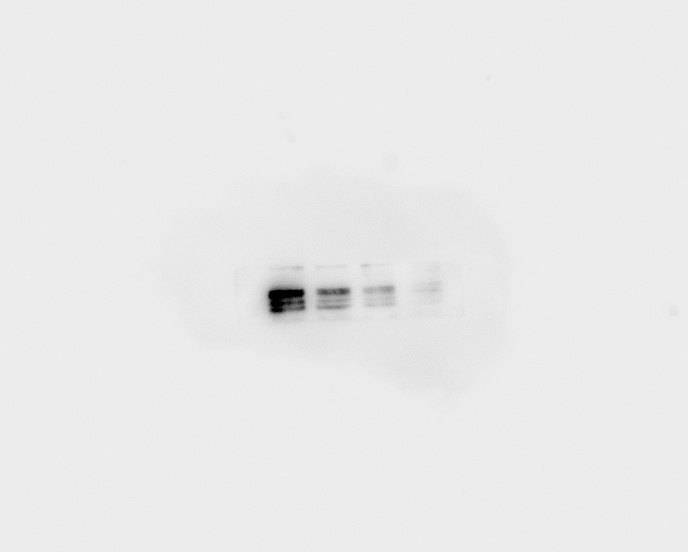

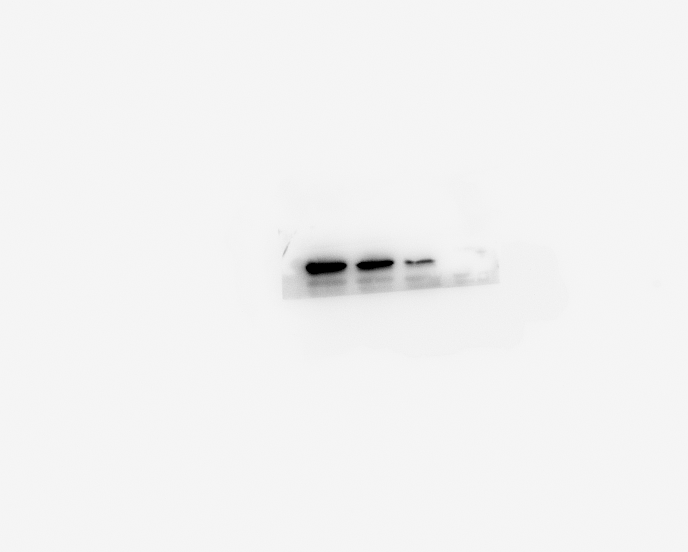

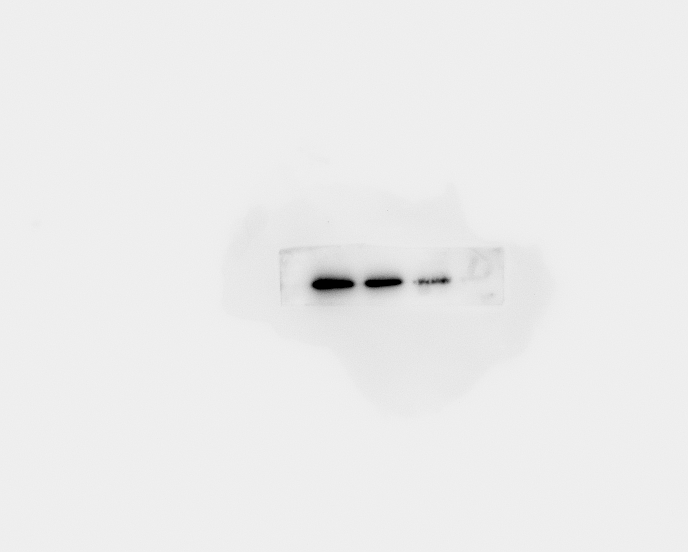

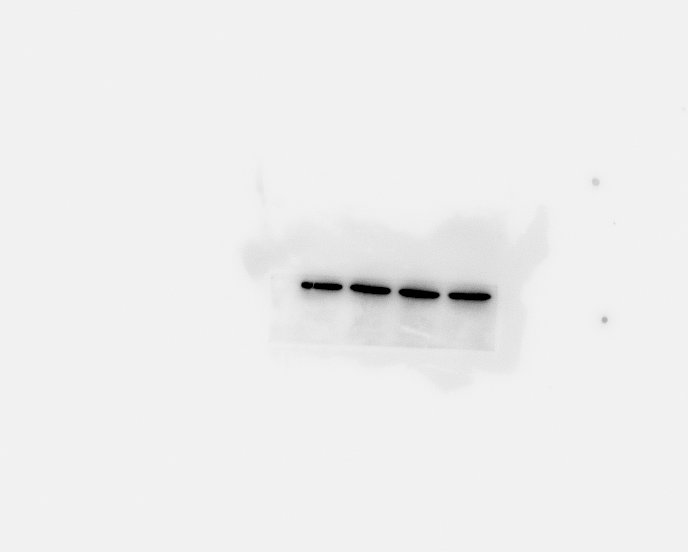


Western blot raw data in Fig. 8E


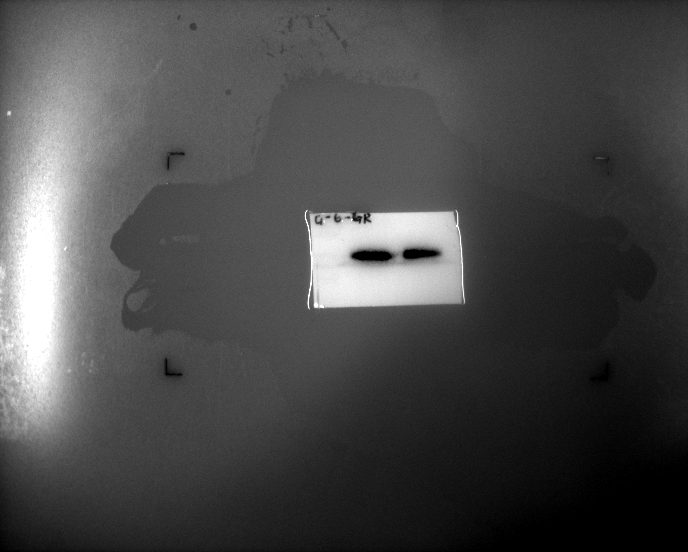

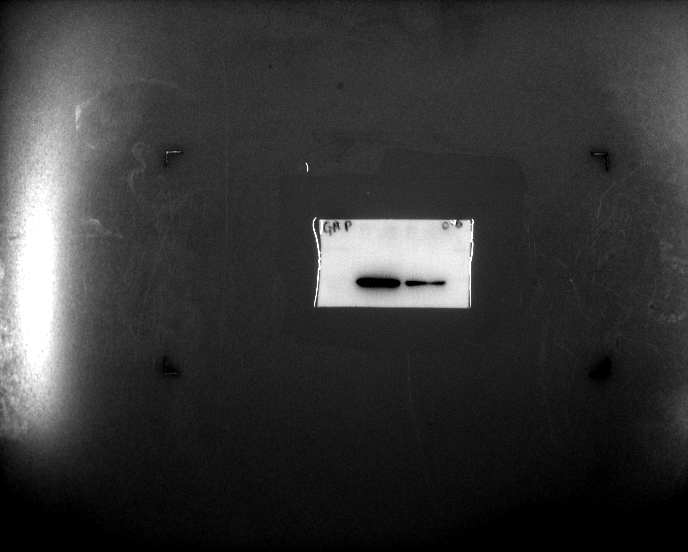

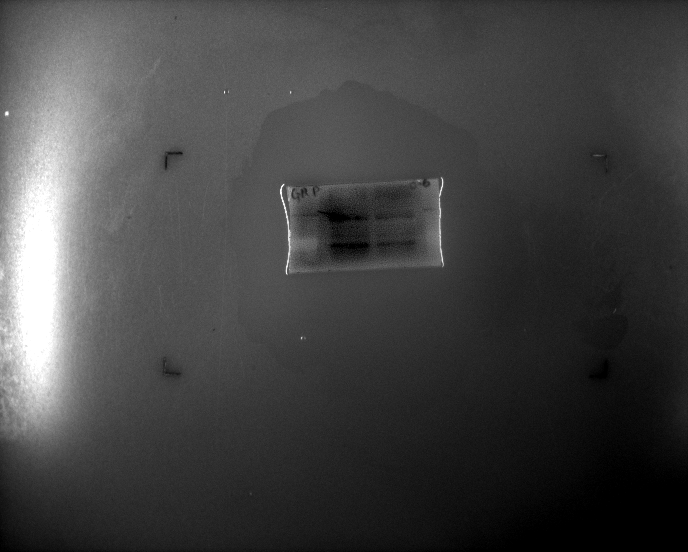


Western blot raw data in Fig. S4A


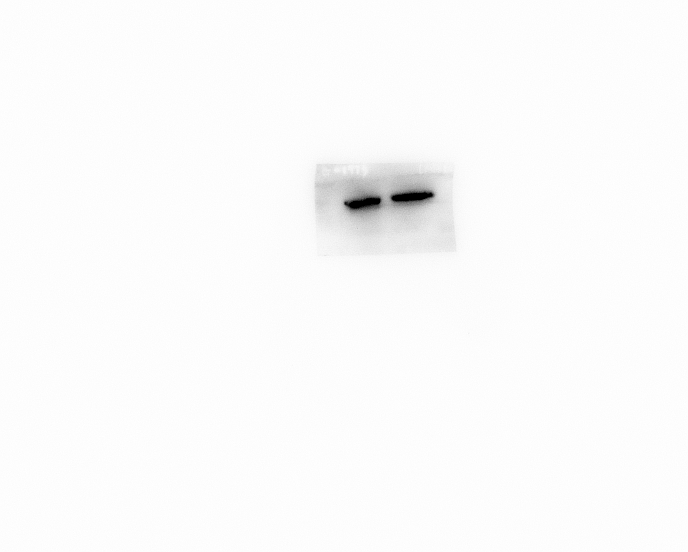

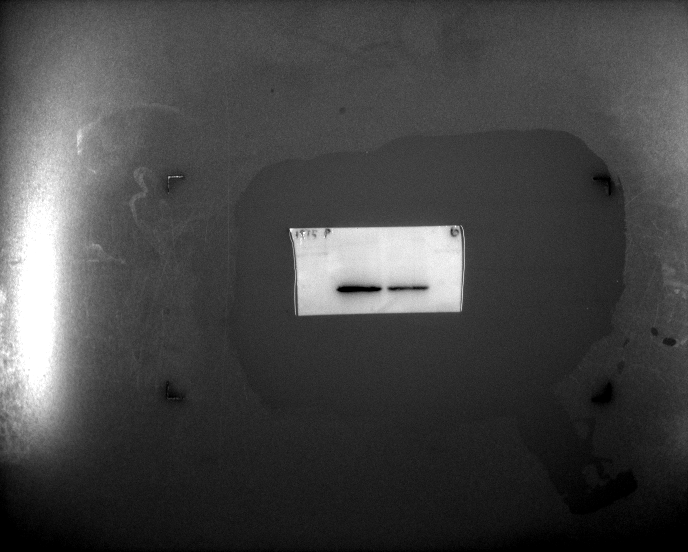

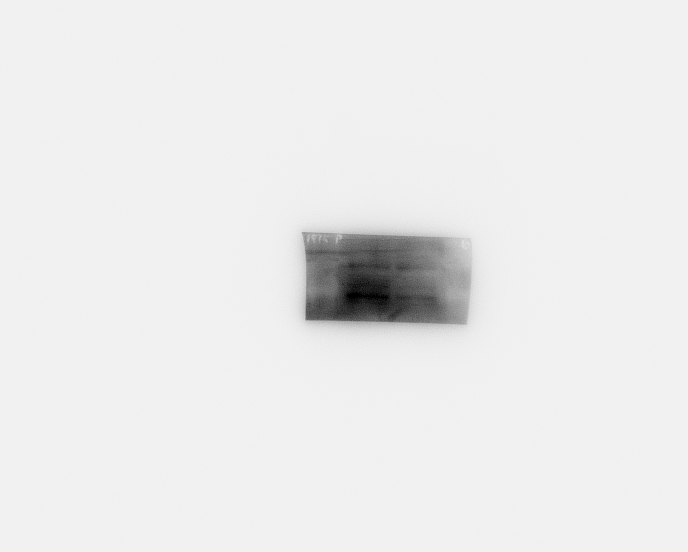


Western blot raw data in Fig. S4B


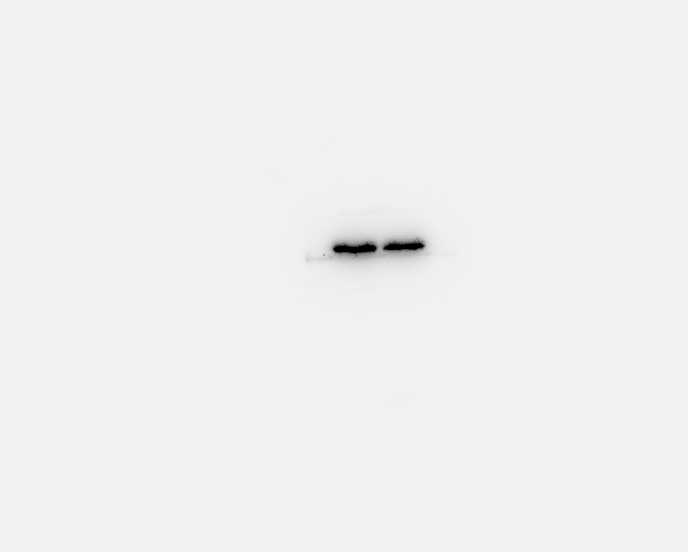

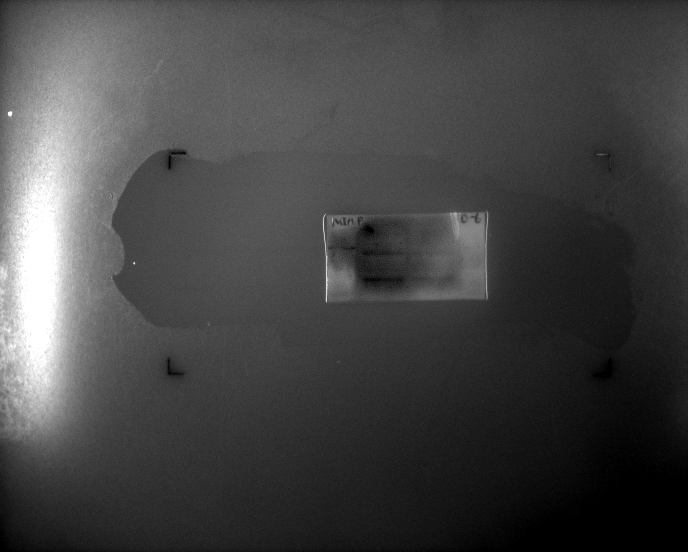

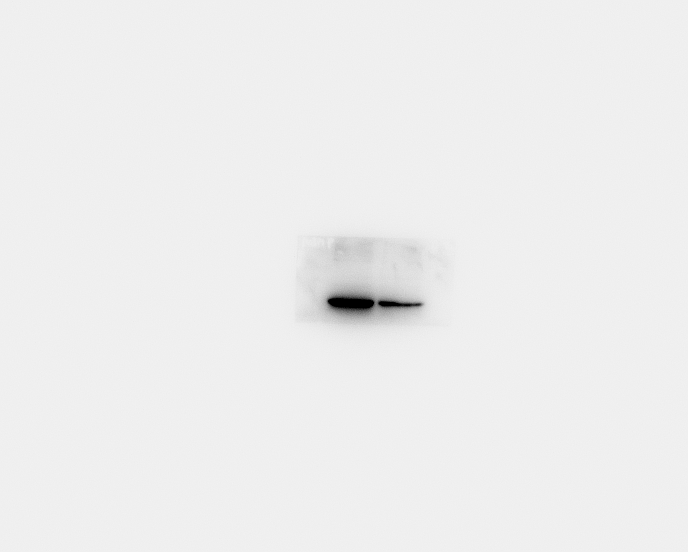


Western blot raw data in Fig. S4C
